# Assembly of a patchy protein into variable 2D lattices via tunable multiscale interactions

**DOI:** 10.1038/s41467-020-17562-1

**Published:** 2020-07-28

**Authors:** Shuai Zhang, Robert G. Alberstein, James J. De Yoreo, F. Akif Tezcan

**Affiliations:** 1 Department of Materials Science and Engineering, University of Washington, Seattle, WA 98195 USA; 2 Physical Sciences Division, Pacific Northwest National Laboratory, Richland, WA 99352 USA; 3 Department of Chemistry and Biochemistry, University of California, San Diego, La Jolla, CA 92093 USA; 4 Materials Science and Engineering, University of California, San Diego, La Jolla, CA 92093 USA

**Keywords:** Atomic force microscopy, Biophysics, Surface assembly, Biomaterials, Nanoscale materials

## Abstract

Self-assembly of molecular building blocks into higher-order structures is exploited in living systems to create functional complexity and represents a powerful strategy for constructing new materials. As nanoscale building blocks, proteins offer unique advantages, including monodispersity and atomically tunable interactions. Yet, control of protein self-assembly has been limited compared to inorganic or polymeric nanoparticles, which lack such attributes. Here, we report modular self-assembly of an engineered protein into four physicochemically distinct, precisely patterned 2D crystals via control of four classes of interactions spanning Ångström to several-nanometer length scales. We relate the resulting structures to the underlying free-energy landscape by combining in-situ atomic force microscopy observations of assembly with thermodynamic analyses of protein-protein and -surface interactions. Our results demonstrate rich phase behavior obtainable from a single, highly patchy protein when interactions acting over multiple length scales are exploited and predict unusual bulk-scale properties for protein-based materials that ensue from such control.

## Abstract (web summary)

As nanoscale building blocks, proteins offer unique advantages, including monodispersity and atomically tunable interactions, but their self-assembly is limited compared to inorganic or polymeric nanoparticles. Here, the authors show modular self-assembly of an engineered protein into four physicochemically distinct patterned 2D crystals via control of four classes of interactions.

## Introduction

Self-assembly provides a powerful means to organize matter at all length scales and is responsible for the emergence of living systems from inanimate nanoscale objects^1^. For example, although discrete proteins generally adopt well-defined structures, assembly into higher-order architectures considerably broadens the scope and complexity of their capabilities to fulfill the stringent requirements of life. Synthetically, even hard, noninteracting nanoparticles can spontaneously arrange into close-packed structures to maximize system entropy^2,3^. Yet, as component complexity increases through the introduction of specific interactions and anisotropy (i.e., patchiness)—so does the resulting structural and functional diversity^3–5^.

As patchy nanoparticles, proteins present several advantages: they are chemically monodisperse and can be tailored with atomic precision at different levels to control assembly: locally (e.g., through discrete chemical bonds)^6,7^, regionally (e.g., through associative noncovalent patches or charged surfaces)^8,9^, or globally (e.g., through shape complementarity and overall charge)^10,11^. Yet, the structural and chemical complexity of proteins also renders energy landscapes controlling their self-assembly highly intricate, with many minima of similar energies separated by small barriers^12,13^. This characteristic is universally exploited by living systems to create dynamic and reconfigurable architectures whose structures can be altered by environmental conditions^14^ or physical templates (e.g., chaperones^15^ and 2D membranes^16^), but translates to poor predictive control in synthetic systems. Accordingly, design methodologies for artificial protein assemblies have primarily focused on introducing a single type of enthalpy-driven interaction^6,8,17,18^ to circumvent this complexity. Consequently, self-assembled products have generally been singular in that one building block leads to one assembled structure, residing in a deep energy well and exhibiting little dynamic or responsive behaviour^19,20^ unless the designed interactions themselves are inherently flexible or tunable^6,7,21,22^. In other words, the patchiness of proteins has not been fully exploited to date, despite offering the opportunity to create multipurpose synthetic building blocks whose assembly can be directed toward a diverse set of outcomes, provided the relative depths of the free-energy minima (and corresponding barrier heights) can be manipulated.

2D materials are particularly attractive as targets for directing diverse assembly outcomes because they enable use of surfaces as templates (whose physical and chemical properties can be systematically varied) to modulate the free-energy landscape across which assembly takes place. Here we report the experimental realization of complete synthetic control over protein self-assembly pathways, whereby a singular patchy protein is arranged into alternate 2D crystalline structures through modulation of its surface templated self-assembly via environmental conditions. Our results demonstrate that the concept of patchiness can be adapted to protein design by considering the magnitudes and length scales of different interactions.

## Results

### AFM characterization of solution-assembled ^C98^RhuA crystals

**Fig. 1:**
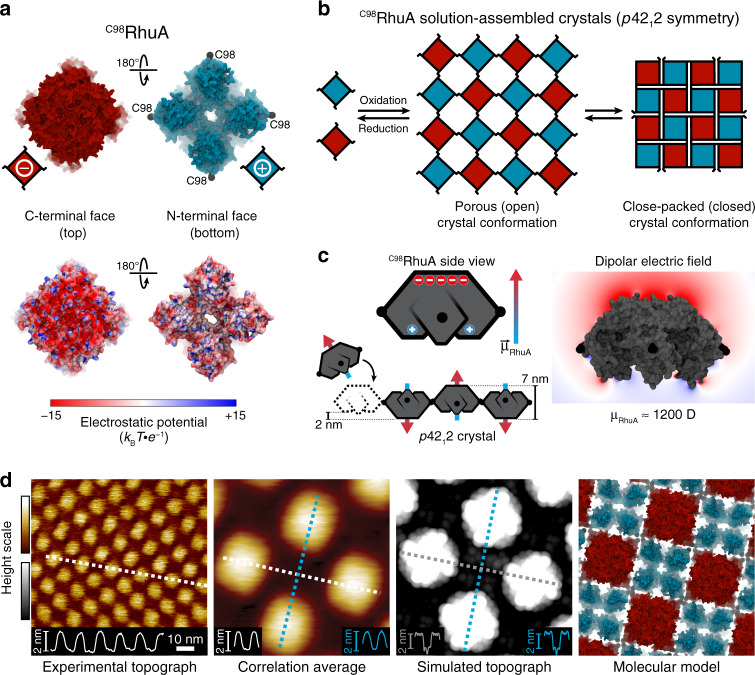
Solution self-assembly of ^C98^RhuA. **a** Solvent-excluded surfaces and electrostatic potential maps of ^C98^RhuA termini faces, colored according to charge. Depth cueing is used to highlight the two distinct topographies of each terminus. C98 residues are shown as black spheres. **b** Schematic depicting ^C98^RhuA solution self-assembly into 2D *p*42_1_2 crystals and their emergent auxetic behavior. **c** Origin of the ^C98^RhuA macrodipole moment and its global-scale electric field (red to blue color range: −7.5 to +2.5 *k*_B_*T* ∙ *e*^−1^). Cartoon arrows represent the dipole orientation of each protein. **d** High-resolution AFM topographs and corresponding structural model of solution-assembled close-packed crystals. The height profiles correspond to the dashed lines. Height ranges (gradient): 5 nm (experimental), 2 nm (simulated).

We utilized the highly patchy protein building block L-rhamnulose-1-phosphate aldolase, a *C*_4_-symmetric protein modified at its corners with Cys residues (^C98^RhuA) (Fig. 1a), which we previously showed self-assembles in solution into μm-sized, 2D crystals via intermolecular disulfide bonds and 3D crystals through stacking of the layers^7^. While disulfide bond reversibility enabled defect-free self-assembly under thermodynamic control, bond flexibility endowed the 2D lattices with coherent dynamic behaviour, whereby the lattices opened and closed via correlated in-plane rotations of ^C98^RhuA molecules (Fig. 1b)^7,23^. Interestingly, transmission electron microscopy and electron diffraction analyses suggested that the lattices exclusively possessed *p*42_1_2 symmetry^7^, whereby neighbouring ^C98^RhuA molecules adopted an out-of-plane antiparallel (up-down) arrangement. Atomic force microscopy (AFM) imaging of solution-assembled ^C98^RhuA crystals directly confirms this periodic, alternating arrangement of ^C98^RhuA molecules throughout individual lattices (Fig. 1d). The lower protein layer is most clearly resolved at step edges, where tip-protein convolution artefacts on the terrace are minimized (Supplementary Fig. 1).

### Dipolar interactions mediate molecular patterning of ^C98^RhuA

Given that the only interprotein connections in the ^C98^RhuA lattices are disulfide bonds^23^ (all protein-protein contacts are >5 Å, which is too distant even for weak hydrogen bonds, salt-bridging, or hydrophobic contacts), we originally attributed this distinct up-down arrangement to an energetic bias imposed by differences in disulfide bond conformational strain resulting from different protein orientations^7^. However, intuition would instead suggest that the flexibility of the disulfide hinges should prevent any such substantial bias. Therefore, we carried out all-atom molecular dynamics (MD) simulations, which indeed showed that the conformational energies of disulfide bonds are averaged out within the 2D ^C98^RhuA lattice regardless of symmetry (Supplementary Fig. 2). This finding implies there must be indirect inter-protein interactions acting over several nm’s (>5 nm’s) to yield the alternating ^C98^RhuA registry.

We next considered the possible impact of dipole-dipole interactions, which have been shown to promote antiparallel packing in covalent organic frameworks^24^, small-molecule crystals^25^, and nanoparticle lattices^26–28^, and have been invoked in stabilization of biomolecular complexes^10^, but never implicated as a determinant of protein self-assembly. Examination of the ^C98^RhuA structure reveals a highly anisotropic charge distribution, with the flat C-terminal top and four-legged N-terminal bottom being negatively and positively charged, respectively (Fig. 1a, c), yielding a sizeable macrodipole moment (*ca*. 1200 D), which is greater than those of 90–95% of proteins^10^. This polarized structure allows ^C98^RhuA to be effectively modelled as a physical dipole (Fig. 1c, Supplementary Discussion), as done previously for electrostatically anisotropic inorganic nanoparticles^26,28,29^. Calculation of the pairwise ^C98^RhuA dipole-dipole interaction over 7–10 nm (dimensions accessible by lattice conformations) reveals a smooth, funnel-shaped energy landscape invariably favoring the antiparallel arrangement of *p*42_1_2 crystals—relative to parallel—by 0.13–1.11 kcal∙mol^−1^ (0.22–1.87 *k*_B_*T*), even at such long distances (Supplementary Fig. 3). Despite this energy being on the order of weak noncovalent bonds, its magnitude is rapidly amplified fourfold (up to 4.2 kcal∙mol^−1^ or 7 *k*_B_*T*) by nearest-neighbour interactions (Supplementary Fig. 4), propagating *p*42_1_2 symmetry to newly incorporated monomers during solution-phase crystallization. This strongly suggests that the collective effect of a multitude of weak, long-range, orientation-dependent interactions may play an important role in controlling the precise 2D, nanoscale patterning of ^C98^RhuA assemblies. In addition, it was previously postulated based on a statistical analysis of protein crystals that space- or plane-group symmetries that offer more degrees of configurational freedom would be preferentially populated over those that offer less (e.g., *p*42_1_2 over *p*4)^30,31^. Thus, in the case of ^C98^RhuA self-assembly in solution, *p*42_1_2 symmetry is both statistically and thermodynamically (through dipolar interactions) preferred over *p*4 symmetry. Determining the relative energetic contributions of these effects and predictably engineering them to modulate protein self-assembly over different length scales represent an exciting avenue for future studies.

### Surface-templated self-assembly of ^C98^RhuA crystals

**Fig. 2:**
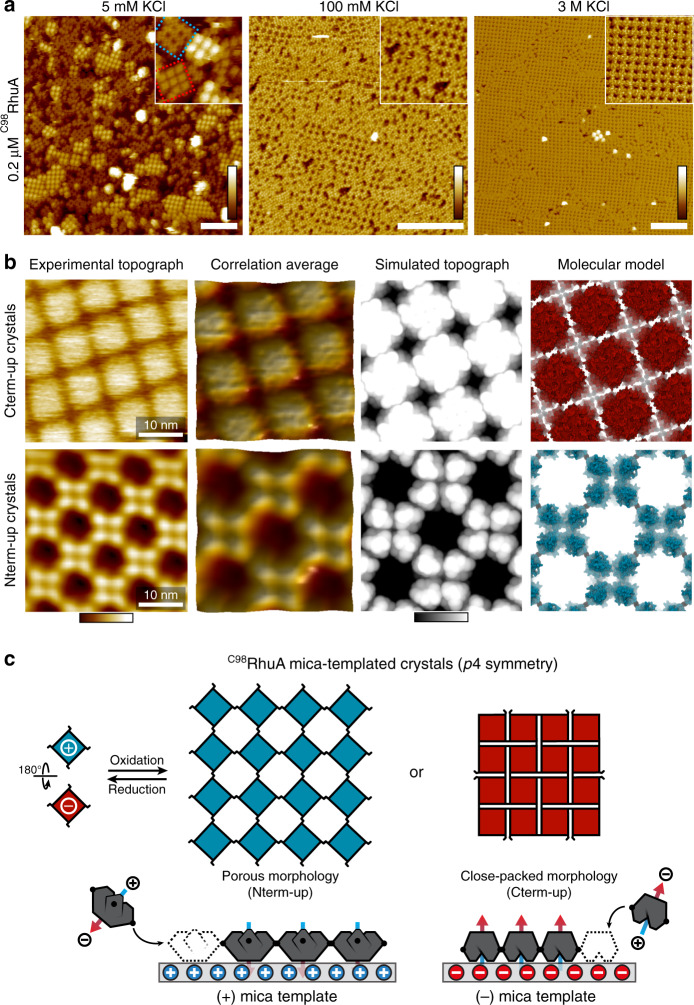
Surface-templated self-assembly of *p*4 ^C98^RhuA crystals. **a** Growth of monolayer ^C98^RhuA crystals on *m*-mica under increasing [K^+^]. Scale bars (white) are 100 nm. Height ranges (gradient): 20 nm, 10 nm, 10 nm (left to right). **b** High-resolution AFM topographs and structural models of mica-templated crystals. Height ranges (gradients): 7 nm (experimental), 3 nm (simulated). **c** Cartoon overview of mica-templated self-assembly. Blue and red colors represent positive and negative charges, respectively.

The above findings suggest the charge anisotropy of ^C98^RhuA building blocks and their propensity to form 2D lattices might be exploited to control their assembly by using surfaces that can modulate the balance of forces. As a template, we used muscovite mica (*m*-mica) (001): a pseudo-hexagonal tessellation of negatively charged cavities occupied by native K^+^ ions or other cations at a coverage that can be varied to tune the net surface charge^32^. We found that ^C98^RhuA readily formed large domains of 2D square lattices on *m*-mica at ≥750-fold lower concentrations than required for solution assembly (≤0.2 μM vs. ≥150 μM). These lattices exhibited both open and closed pore conformations resembling those of solution-grown crystals, indicating the designed intermolecular disulfide bonding was preserved and that ^C98^RhuA*-m*-mica interactions were mediated by the charged surfaces of ^C98^RhuA (Fig. 2). Indeed, a variant in which Cys98 residues were replaced with Ser (^S98^RhuA) adsorbed to *m*-mica but failed to form 2D lattices even at concentrations of 50 μM (Supplementary Fig. 5). ^C98^RhuA assembly on *m*-mica could also be prevented (reversed) in the presence of 1 mM reductant, which prevents (reverses) disulfide bonding (Supplementary Fig. 6).

In contrast to the antiparallel registry of solution-assembled lattices, crystals formed on *m*-mica possessed *p*4 symmetry, whereby neighbouring proteins adopted a parallel arrangement, despite the repulsive dipole-dipole interactions, demonstrating that protein-*m*-mica interactions could selectively template alternative crystal structures (Fig. 2). In 5 mM KCl, two classes of *p*4-symmetric ^C98^RhuA crystals formed concurrently: close-packed crystals with C-terminus up (Cterm-up crystals) and porous crystals with N-terminus up (Nterm-up crystals) (Fig. 2a, red and blue dashed boxes). As the resolution of the AFM images were sufficient to resolve the central dip between the N-terminal lobes of individual ^C98^RhuA molecules (Supplementary Fig. 7), the relative orientations of the adsorbed proteins were readily determined from their surface topologies (Figs. 1a, and 2b). Interpretations of the images were corroborated by simulated surface profiles calculated from structural models of *p*4-symmetric ^C98^RhuA crystals (Fig. 2b). The distribution, size, and degree of order of these two crystal classes could be tuned via the KCl concentration: At 5 mM K^+^, both Nterm- and Cterm-up crystals formed (Fig. 2a, left). At 100 mM K^+^, poorly ordered Nterm-up crystals dominated (Fig. 2a, middle) and at 3 M K^+^, self-assembly exclusively yielded highly ordered 2D Nterm-up lattices containing hundreds to thousands of monomers (Fig. 2a, right).

This variability in ^C98^RhuA assembly can be understood from the charge state of *m*-mica (Fig. 2c), which follows a well-documented trend towards positive values with increasing KCl^32^. Freshly cleaved *m*-mica surfaces are negatively charged in neutral aqueous solution. The 5 mM K^+^ is insufficient to occupy all negatively charged sites on the surface and the cations are distributed heterogeneously, preferentially forming extensive domains of negative and positive charge^33^. At [K^+^] > 100 mM, the surface sites are more than 50% occupied, fully compensating the surface charge and resulting in positive zeta potentials^32,34^. Accordingly, while 0.2 μM solutions of ^C98^RhuA yielded a mixture of Cterm-up and Nterm-up crystals at 5 mM K^+^, only Nterm-up crystals were observed at >1 M K^+^ (Fig. 2a). At much lower [^C98^RhuA], Cterm-up crystals formed exclusively at [K^+^] ≤ 5 mM, indicating preferential attachment to the negatively charged bare mica surface; this morphology was never observed at [K^+^] > 1 M (Supplementary Fig. 8). Moreover, selectivity for monomer/crystal binding orientation followed well-known trends in specific ion adsorption to *m*-mica:^32,35^ low-affinity counterions (e.g., Zn^2+^) only produced Cterm-up crystals, while Rb^+^—a known substitute for K^+^^36^—and Mg^2+^ (widely used to induce surface-charge reversal on *m*-mica for DNA adsorption^37^) recapitulated the behaviour with K^+^ (Supplementary Fig. 9).

### Hierarchical self-assembly of bilayer ^C98^RhuA crystals

**Fig. 3:**
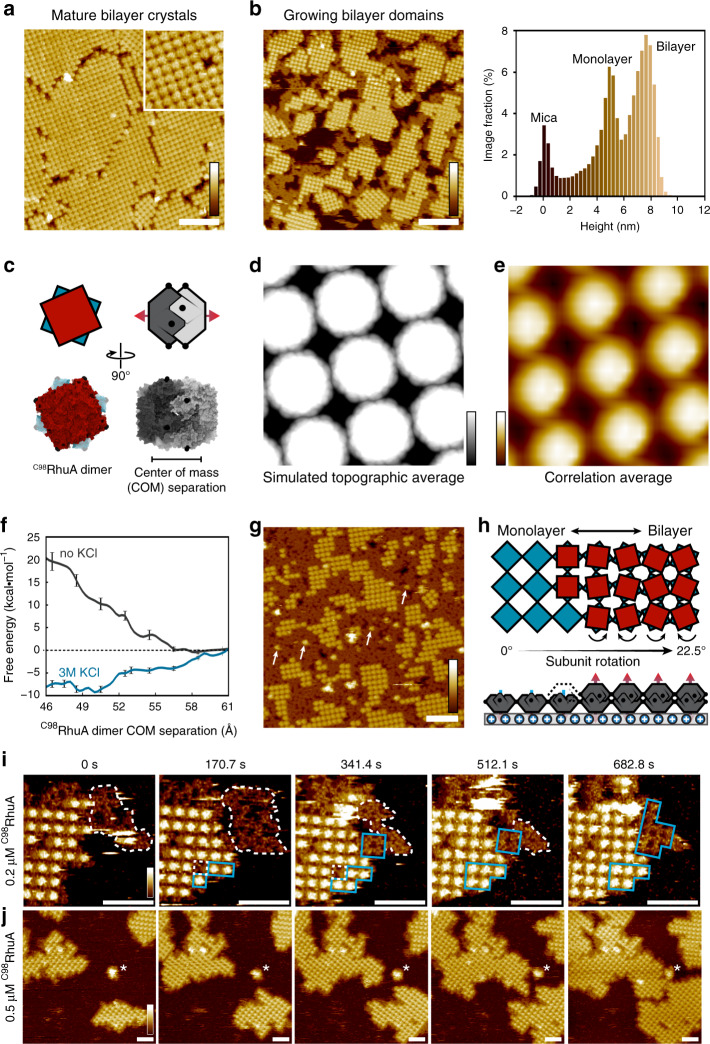
Hierarchical self-assembly of bilayer ^C98^RhuA crystals. High-resolution AFM images of **a**, mature and **b**, growing bilayer crystals (0.4–0.5 μM ^C98^RhuA), with image height value histogram shown on right. **c** ^C98^RhuA dimer structure. **d** Simulated and **e**, experimental average topographs for the bilayer structure. **f** Potential of mean force for ^C98^RhuA dimerization in the presence/absence of 3 M KCl. Error bars: s.e. of the free-energy gradient, as calculated using the block-averaging method (“Methods”). **g** Self-assembly of 5 μM ^C98^RhuA, resulting in disordered monolayers which cannot template bilayer domains. Isolated 2nd layer monomers (arrows) are rare outside of crystalline domains. **h** Proposed self-templated growth mechanism for bilayer crystals. **i** N-term up ^C98^RhuA monolayers grow via nonclassical amorphous (white contour)-to-crystalline (blue regions) transitions at edges, which subsequently template second layer growth through classical (monomer-by-monomer) addition. **j** Bilayer growth kinetics are limited by the growth rate of the first layer. Scale bars (white): 100 nm (**a**/**b**/**g**), 50 nm (**i**/**j**). Height ranges (gradient): 10 nm (**a**/**b**/**j**), 2 nm (**d**), 4 nm (**e**), 12 nm (**g**), 8 nm (**i**).

When [^C98^RhuA] was increased to >0.2 μM and [K^+^] was held at 3 M, a third 2D lattice arrangement with a height of 9 nm emerged (Fig. 3). The surface topography resembled an array of Cterm-up monomers (Fig. 3a), but this array formed exclusively atop domains of 2D Nterm-up crystals (Fig. 3b, left) and was shorter than twice the height (*ca*. 10 nm) of the underlying Nterm-up monolayer (Fig. 3b, right), implying the ^C98^RhuA monomers in each layer must interact via interpenetration of the corrugated N-term faces (Fig. 3c). We hypothesized that this tail-to-tail packing is isostructural with the obligate, 9.5-nm tall dimers of the ^F88/C98^RhuA variant, whose formation during growth of crystals in bulk solution is stabilized by hydrophobic, Phe88-mediated interactions between the N-term faces^7^. Indeed, the simulated topography for a bilayer based on the ^F88/C98^RhuA lattice structure (Fig. 3d, Supplementary Fig. 10) closely resembles the high-resolution AFM images (Fig. 3e). Importantly, the unique circular shape of the protein units in bilayer crystals seen by AFM is reproduced upon averaging of both substructures of the ^F88/C98^RhuA lattice (Supplementary Fig. 10). Furthermore, free-energy (i.e., potential of mean force) calculations show that ^C98^RhuA Nterm-Nterm intercalation is weakly favoured in the presence of 3 M KCl due to preferential hydration of ions (i.e., by salting out) and protein shape complementarity, but highly disfavoured at low ionic strength (Fig. 3f). N-term intercalated ^C98^RhuA dimers can therefore be populated only when incorporated into the matrix of the bilayer, wherein they self-associate via lateral disulfide bonds to form the second layer. As expected from the weak interlayer interactions combined with the strong in-plane disulfide bonds, lowering the ionic strength ([KCl] = 100 mM) after the formation of bilayers at high salt yielded free *p*4-symmetric monolayer lattices (likely through exfoliation) (Supplementary Fig. 11) that cannot form without the template.

The importance of disulfide bonding is reflected by the rarity of isolated 2nd layer monomers (i.e., N-term intercalated dimers) (Fig. 3g). If adsorbed randomly with no energetic bias for adsorption next to neighbouring proteins, the expected coverage of isolated monomers for the total 2nd layer coverage seen here (23%) would be 7%^38^, which is 20 times the observed 0.35% coverage of isolated monomers (Supplementary Fig. 12). Thus, the bilayer morphology is realized via a self-templating effect by the underlying *p*4-symmetric monolayer, whose subunits necessarily undergo a 22.5° rotation to bring the monomers in the second layer into the proximity required for disulfide bonding (Fig. 3h, Supplementary Fig. 10). All-atom MD simulations carried out in 3 M KCl show that the C-termini of *m*-mica-adsorbed ^C98^RhuA molecules preferentially reside 8–9 Å away from the mica surface (above the ionic slipping plane) (Supplementary Fig. 13), suggesting a negligible energetic barrier to in-plane rotation.

Seeking deeper insight into the bilayer formation mechanism, we used *in-situ* AFM to follow assembly and found two different growth mechanisms acting concurrently. At 0.2 μM [^C98^RhuA], the first (Nterm-up) layer crystallized via a nonclassical two-step nucleation process^39,40^, whereby the growing edges (Fig. 3i) advanced by initial formation of amorphous regions (white dashed contour), which then spontaneously crystallized (blue dashed square). (Interaction with the adjacent nucleus that has already crystallized may catalyse the transition, a behaviour that has been observed previously during surface crystallization of S-layer proteins^13^.) Additionally, the *m*-mica lattice likely assists in the transition, as evidenced by the alignment of crystal domains with specific *m*-mica (001) lattice directions (Supplementary Fig. 14), which must be imposed at the time of the transition. This capability for reorganization and ordering implies relatively weak ^C98^RhuA-K^+^ surface interactions and is likely facilitated by the reversibility of disulfide bonds.

Unlike the first layer, the second layer grew via a classical mechanism with single monomers binding to existing crystalline monolayers (Fig. 3i, j). At much higher [^C98^RhuA] of 5 μM, the *m*-mica surface became fully covered by the amorphous layer before crystallization could occur, leaving the film kinetically trapped in a disordered state (Fig. 3g). Consequently, only small bilayer domains formed and were surrounded by an amorphous monolayer (Supplementary Fig. 12).

The ability of the surface-templated crystalline monolayer to further enable epitaxial growth of a second layer and the emergence of a stepwise self-assembly mechanism demonstrates how the balance of relative interaction energies can modulate assembly pathways to obtain a specific thermodynamic product, while modulating the protein flux via solution concentration can, instead, select for kinetic products^41^. Such tunability underscores the principal advantage of using proteins as patchy building blocks for self-assembled materials.

### Diverse morphologies from a singular patchy protein

**Fig. 4:**
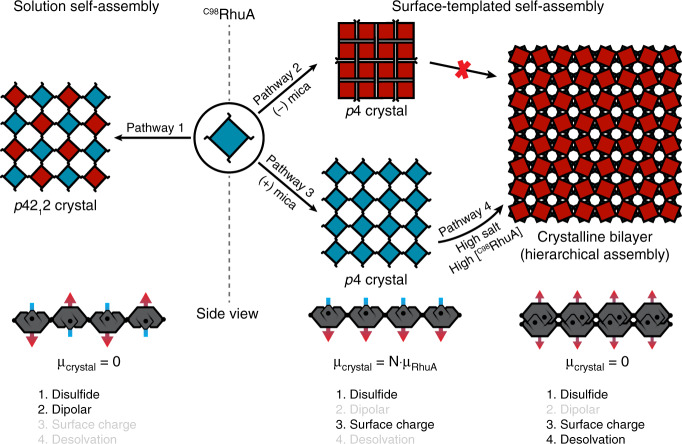
Self-assembly pathways afforded by ^C98^RhuA patchiness and template effects. Overview of all ^C98^RhuA growth pathways and the self-assembly interactions responsible for their formation. The configuration-dependent dipole moment of each crystal morphology is also indicated.

In contrast to our previous work^7^, which described the solution self-assembly of different RhuA variants into distinct 2D crystals (i.e., three building blocks—three assemblies), our findings here (Fig. 4) demonstrate that ^C98^RhuA can be exploited as a singular patchy particle capable of forming multiple morphologies owing to a multiplicity of tunable interactions that govern self-assembly. Key to all four observed morphologies and their attendant pathways are strong but reversible disulfide bonds (operating at a local level) that ensure self-assembly into 2D crystals. Untemplated solution self-assembly through Pathway 1 requires high protein concentrations and leads to the antiparallel out-of-plane registry of ^C98^RhuA. This arrangement likely is a consequence of weak interactions between neighbouring macrodipoles (operating at a global level) in addition to the anticipated statistical preference of *p*42_1_2 symmetry over *p*4^30^. In Pathways 2 and 3, inclusion of the charged mica template drastically lowers the protein concentration needed for self-assembly due to favourable electrostatic interactions between *m*-mica and anisotropically charged ^C98^RhuA. These interactions (at a regional level) are strong enough to overcome the dipole-dipole interactions between ^C98^RhuA molecules, forcing a parallel in-plane registry, while alternating the mica surface charge affords control (also at a global level) over the absolute crystal orientation. Upon increase of ionic strength and protein concentration in Pathway 4, favourable desolvation (regional) interactions become operative between the shape-complementary N-term faces of ^C98^RhuA, leading to formation of bilayer crystals. Such rich phase behaviour from a single building block is rare in nanoparticle self-assembly^4^ and unique among self-assembled protein lattices—natural or designed—because intermonomer interaction surfaces in such lattices are too extensive to allow reconfigurability.

### Emergent functional properties of template-grown p4 crystals

**Fig. 5:**
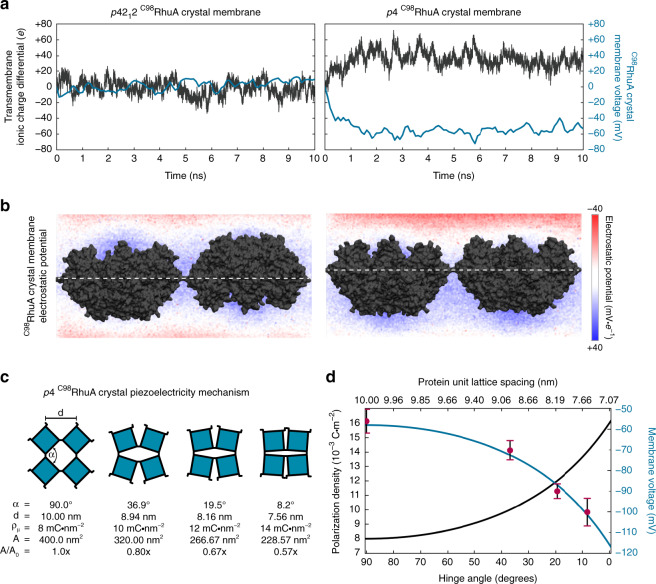
*p*4 ^C98^RhuA crystals are electrets and piezoelectric materials. **a** Net charge imbalance due to ion conduction across the disulfide bonds (half of the simulation box) and resulting electric potential difference across open-state crystals. Dipole-less *p*42_1_2 crystals induce negligible charge separation, while *p*4 crystals generate a steady-state 50–60 mV potential difference across the lattice (membrane voltage). **b** Volumetric slices of the electrostatic potential across the central plane of each system directly illustrate the polarization. **c** Piezoelectric properties of *p*4-symmetry ^C98^RhuA crystals, arising from mechanical coupling between the polarization density (ρ_μ_) and lattice conformation (left). The magnitude of this effect was predicted analytically (right) from the ρ_μ_ over all lattice conformations (as defined by the rotating squares model^7^), while the points reflect numerically determined voltages from all-atom simulations at discrete conformations (Supplementary Fig. 15). The associated hinge angle (α), lattice spacing (**d**), ρ_μ_, and absolute (A) and relative (A/A_0_) unit cell areas for each point are indicated at left. Error bars represent the s.d. of the membrane voltage over the last 5 ns of simulation.

Varying the arrangement of molecules or nanoparticles within a material is known to give rise to different emergent bulk-scale properties^42,43^. Consequently, we considered the effect of the dipolar interactions within the surface-templated 2D phases on their properties. ^C98^RhuA crystals with *p*4-symmetry (one of only 10 polar crystal classes) contain an extended array of coaligned dipoles (Fig. 4) and are formally electrets: polarized dielectric materials possessing a permanent electric field^44^. Although electrets are metastable configurations typically manufactured via thermal annealing in the presence of a strong field, they were achieved in this case through surface-template effects and the strength/topology of the covalent disulfide bonds that preserve structure after assembly. The resulting parallel configuration of the dipoles is indeed predicted to confer unique properties: all-atom MD simulations of infinitely-periodic open-state *p*4-symmetric ^C98^RhuA crystals immersed in a 200-mM NaCl solution predict that Na^+^ and Cl^−^ ions will rapidly segregate across the lattice, moving against their electrochemical gradients until achieving steady-state within 2–3 ns. Importantly, such behaviour is not observed for *p*42_1_2-symmetric crystals (Fig. 5a), consistent with their nonpolar crystal classification (Fig. 5b). At equilibrium, a charge differential of 0.08–0.1 *e* ∙ nm^−2^ was attained across the membrane, corresponding to a reversal potential of 50–60 mV (Fig. 5a). Both the charge differential (40 *e*) and membrane potential are in excellent agreement with analytical calculations (Supplementary Discussion) based exclusively on the density of permanent electric dipoles within the crystal (polarization density), indicating that the effect can be treated as purely dipolar in nature.

Given that the polarization density (0.008–0.016 C ∙ m^−2^ depending on crystal conformation, Fig. 5c) and resulting electric field strength is intrinsically coupled to in-plane compression and expansion, *p*4-symmetric ^C98^RhuA crystals are thus expected to exhibit piezoelectric properties^44^, while *p*42_1_2-symmetric crystals are not. Subsequent all-atom simulations directly confirmed this prediction, achieving density-dependent reversal potentials of ≥100 mV at sufficiently compressed conformations (Fig. 5c, Supplementary Fig. 15). This example illustrates the prospect of obtaining specific bulk-scale physical properties through precise control over the nanoscale patterning of protein building blocks, which in turn can be designed at the molecular level. Polarized membrane materials such as *p*4-symmetric ^C98^RhuA crystals should also be promising candidates for applications in biological nanoelectromechanical systems, including as nanogenerators and passive charge-storage devices, with tunable electrical properties coupled to mechanical in-plane lattice compression.

## Discussion

2D materials have garnered much interest due to their unique physical and chemical properties and applications as membranes, molecular displays and templates for inorganic or biological patterning^45–47^. In these contexts, reconfigurable systems like ^C98^RhuA lattices offer distinct advantages in that the densities and patterns can be facilely altered without the need to engineer multiple building blocks. Unlike previous work, such as our own that reported the synthesis of singular structures (*p*42_1_2 crystals) from a single protein^7^, the findings presented above demonstrate that ^C98^RhuA represents a patchy particle that is pleomorphic—i.e., it is capable of forming multiple structures from a single building block—a largely unexplored phenomenon for engineered proteins.

Our findings reinforce general design principles for patchy particle self-assembly derived from Monte Carlo simulations^41,48^ and underscore the importance of the relative strengths and length scales of interactions over their specific nature in determining the outcome of self-assembly. This substantiates previous analogies between proteins and anisotropic nanoparticles^49,50^ by demonstrating that this conceptual framework is not limited to the latter, but instead represents a generalizable phenomenon for obtaining a diversity of self-assembly outcomes from nanoscale objects^4^. The results also highlight the potential ability of macrodipole moments to influence protein self-assembly at extended length scales (>5 nm), provided they are not overpowered by short-range protein-protein contacts and the system in hand has access to multiple conformational states through flexible interactions. Thus, dipolar forces may represent a powerful addition to the toolkit for designing extended protein-based materials, although systematic studies will be required to firmly establish their magnitude and their general applicability. Properly exploited, the unique sensitivity and angular dependence of polarized building blocks (such as ^C98^RhuA) should permit the use of external fields to direct their molecular-scale organization in solution, potentially enabling the template-free self-assembly of electrically anisotropic biomaterials.

Abstracting from the details of the interactions specific to ^C98^RhuA, we propose three general guidelines for the rational design of pleomorphic building blocks: (1) selectivity for particular structures can be achieved by incorporating interactions across local, regional, and global scales, (2) intrinsic structural/molecular properties (e.g., dipole moment and symmetry) may impart sufficient energetic bias to influence self-assembly over nm distances without direct contact, but ultimately (3) interaction flexibility between building blocks (e.g., disulfide bonds) is essential to enable access to alternative configurations.

## Methods

### Protein purification and mutagenesis

^C98^RhuA and ^F88/C98^RhuA were purified following established protocols (see refs. ^7,23^ for alternative descriptions). Briefly, for each protein, a pJ414 plasmid containing the RhuA coding sequence was transformed into BL21 (DE3) *E. coli* cells (New England Biolabs; Catalog #C2527I) via heat-shock, grown to high density in LB + 100 mg∙mL^−1^ ampicillin, overexpressed by overnight 1 mM IPTG induction, pelleted, resuspended in 20 mM Tris-HCl (pH 7.5) + 10 mM β-mercaptoethanol (βME), and lysed by sonication. The resulting solution was clarified by centrifugation (5000 rpm, 15 min), treated with 1.5% Polymin-P, reclarified, and purified via NaCl step gradient on a DEAE gravity column at 4 °C. Peak fractions were pooled and RhuA was precipitated using 1.7 M (NH_4_)_2_SO_4_, gently stirred for 30 min, then separated by centrifugation. The precipitate was dialyzed into 20 mM sodium acetate (pH 5) + 10 mM βME, exchanging 3–4 times over 3 days. The dialysate was sterile filtered, loaded onto S columns via FPLC and purified via NaCl gradient. RhuA elutes at ~200 mM NaCl for both columns. Peak fractions (>90% purity) were pooled prior to concentration and storage.

Overexpression and purification of ^S98^RhuA was carried out analogously to ^C98^RhuA except for the omission of βME in the purification buffers. All purified proteins were dialyzed into 20 mM Tris-HCl (pH 7.5) and 10 mM reduced L-glutathione (GSH), concentrated to 100–150 μM, flash-frozen in liquid nitrogen, and stored at ≤ −60 °C. The plasmid for ^S98^RhuA was generated from the ^C98^RhuA parent plasmid via site-directed mutagenesis using the following primers:

RhuA S98 Forward: GTTAAGGTGGATAGCAGCGGTGCAGGTTACCACATCC.

RhuA S98 Reverse: GGATGTGGTAACCTGCACCGCTGCTATCCACCTTAAC.

### Solution self-assembly of ^C98^RhuA

Crystallization of ^C98^RhuA was induced via hand-thawing of frozen RhuA aliquots, which were then placed on a shaking platform at 4 °C and allowed to mature. Nucleation typically occurred within 3–7 days, and crystals fully matured over 2–3 weeks, consistent with previous reports^7,23^. Crystal suspensions were clarified 2–3x by low-speed (*ca*. 3000 rpm) centrifugation in a benchtop centrifuge followed by replacement of the supernatant with fresh buffer to remove unincorporated proteins. This procedure facilitated the binding of *p*42_1_2 crystals onto the mica substrate, but also increased the population of stacked 2D crystals. The suspension of *p*42_1_2 crystals was diluted to 25 μM using 10 mM Tris-HCl (Sigma-Aldrich) pH 7.0 prior to imaging by AFM. 10 μl of diluted crystal suspension was incubated on polylysine-treated mica for 10 min, then rinsed with 10 mM Tris-HCl buffer prior to imaging. Polylysine-treated mica was obtained by incubating 10 μl polylysine solution (0.01%; Sigma-Aldrich) on freshly cleaved mica for 1 min, rinsing with water, and drying under nitrogen prior to use.

### Mica-templated self-assembly of RhuA

Frozen stock solutions of ^C98^RhuA were thawed at room temperature and diluted to the final concentration with incubation buffer (10 mM Tris-HCl pH 7.0, 1–10 μM GSH, and desired concentration of KCl, RbCl, MgCl_2_, or ZnCl_2_). 100 μl of diluted protein solution was deposited onto freshly cleaved mica and incubated for 24 h in a sealed petri dish at room temperature. The mica surface was rinsed with fresh incubation buffer prior to imaging by AFM. GSH, KCl, RbCl, MgCl_2_, and ZnCl_2_ were purchased from Sigma-Aldrich, nuclease-free water from Ambion, and muscovite mica from Ted Pella.

### Atomic force microscopy

All AFM images of mica-templated and solution-grown ^C98^RhuA crystals were captured in PeakForce Tapping^TM^ mode on a MultiMode^TM^ VIII AFM (Bruker, CA) using HYDRA4V-100NG or HYDRA6V-100NG (AppNANO, CA) probes in the incubation buffer used for each specific sample. The peak-force set-point was continuously adjusted to minimize any possible manipulation or damage from probes. The effective imaging force ranged from 100 to 200 pN, within the typical force range for AFM imaging of biomolecules^51,52^. All offline data processing was done using the SPIP^TM^ software package (Image Metrology, Denmark).

### Simulated AFM topographs

AFM topographs were calculated from atomic models with different monomer configurations (extracted from equilibrated 2 × 2 RhuA crystals; see below) using a custom Tcl script executed within VMD^53^, taking the mica surface to be the plane *z* = 0. Accordingly, crystal models were recentered at (*x* = 0, *y* = 0) and moved along the +*z* axis until the minimum *z* position of the protein Cα atoms was at 0. The probe tip was modeled as a sphere with radius 10.0 Å and center *z*_tip_ at the end of a cone with half-angle 20° and truncated at *z*_cutoff_ (see Supplementary Fig. 16). Parametric equations which define the volume enclosed by this tip shape were determined and used to check for overlaps with any protein heavy atoms within the conical or spherical volumes for a given (*x*, *y*, *z*_tip_). This was critical to capture tip convolution artifacts arising from the finite size of the tip, not simply the tip radius. Tapping was simulated by moving the probe to (*x*, *y*, radius) and increasing *z*_tip_ until the number of overlaps was 0 (or below a given threshold to account for the flexibility of disordered protein loops). The height for that *xy* position was then recorded as (*z*_min_ = *z*_tip_ – radius). This calculation was repeated row-by-row, with the resolution determined by Δ*x* = Δ*y* = 0.50 Å, to generate a 2D array of height values analogous to a true AFM topograph. Output was directly visualized and processed using Gwyddion. This code (and associated utilities/example files) can be accessed at 10.5281/zenodo.3924893.

### Evaluation of the ^C98^RhuA dimerization free-energy landscape

The ^C98^RhuA dimer structure was prepared from the ^A88F^RhuA crystal structure (PDB ID: 2UYU) by performing the corresponding D98C and F88A mutations using PyMOL^54^. PSFGEN^53^ was used to add missing hydrogens and assign atom types. The final dimer structure was centered at the origin with the axis of symmetry aligned along the *z*-axis, then each monomer was translated ±10 Å along the *z*-axis to yield a starting distance between each protein’s center of mass (COM separation) of 65.5 Å. The separated dimer was then solvated with 45,082 water molecules (CHARMM TIP3P) and either neutralized with 32 K^+^ ions (no KCl starting structure) or 2302 K^+^ ions and 2270 Cl^−^ ions (3 M KCl starting structure). The CHARMM36 force field^55^ was used for protein atoms and Joung-Cheatham parameters^56^ were used for monovalent ions. Both systems were minimized for 5000 steps with all protein atoms held fixed, followed by a 5000 step minimization without restraints. Protein Cα atoms were constrained to their starting positions by a 10 kcal∙mol^−1^ ∙ Å^−2^ restraint for equilibration. The systems were equilibrated for 1 ns in the isobaric-isothermal (NPT) ensemble (1 atm, 300 K) with the cross-sectional *xy* proportions held constant, yielding final box dimensions of 104.7 × 104.7 × 145.6 Å. Monomers were then linearly pulled towards each other to a final COM-COM distance of 45.5 Å over 5 ns using a 100 kcal∙mol^−1^ ∙ Å^−2^ moving restraint, with the *xy* coordinates of the Cα remaining constrained to prevent rotation of the monomers. Initial coordinates for umbrella sampling windows were extracted from this pulling simulation and maintained with weaker force constants (see Supplementary Table 1 for details). All windows were equilibrated for 25 ns, of which the last 10 ns were used for calculation of the PMF using the WHAM algorithm^57^. The 100 kcal∙mol^−1^ ∙ deg^−2^ harmonic restraints were employed during sampling to prevent rotation of each monomer about their axis of symmetry in order to preserve their relative orientations from the ^F88^RhuA crystal structure, which simplifies the dimerization coordinate to 1D (COM separation along the *z*-axis). Errors in the free energy estimates were calculated using the block averaging method^58^. PMF simulations were carried out using NAMD 2.12^59^.

### Modelling and simulation of protein binding to *m*-mica

Parameters and initial coordinates for muscovite mica were taken from the INTERFACE force field package^60^. A 5 × 3 *m*-mica supercell with Al substitutions in agreement with ^29^Si NMR data was then tiled to form a 5 × 5 array of dimensions 129.795 × 135.230 × 19.9452 Å. Periodic bonds were reorganized using TopoTools^61^ within VMD to yield a “full-size” single layer of *m*-mica corresponding to a 25 × 15 × 1 supercell of the crystallographic *m*-mica unit cell. Copies of this full-size layer were then translated along multiples of the vector (2.005, 0.000, 19.9452 Å) to yield a “5stack” bulk-like m-mica structure of final dimensions 129.795 × 135.230 × 99.726 Å, which was then merged into a single structure using TopoTools. This system was equilibrated at constant pressure without minimization in a fully-flexible orthorhombic periodic cell for 100 ps using the particle mesh Ewald method for full-system electrostatics. The two outermost layers were then removed from the equilibrated structure to yield the final starting *m*-mica coordinates (appx. 60 Å in height), with all surface vacancies on both sides of the mica occupied by K^+^ ions. These ions were free to exchange with the solution, and at equilibrium 80–85% of sites were occupied by K^+^ ions. This state was also attained if the system was initiated with a bare (K^+^ occupancy = 0) *m*-mica surface and the same numbers of ions in solution, confirming that it reflects thermodynamic equilibrium.

A pre-equilibrated ^C98^RhuA structure was recentered at the origin and solvated in a pre-equilibrated 128.8 × 134.0 × 129.9 Å box (68,667 waters) prior to neutralization with 16 K^+^ ions. An additional 3463 K^+^ ions and 3463 Cl^−^ ions were added, bringing the [KCl] to *ca*. 2.67 M (in 61,725 waters). This system was minimized for 1000 steps with all protein atoms fixed and subsequently equilibrated in the NPT ensemble (1 atm, 300 K) for 1 ns keeping the *xy* area constant. The final box dimensions were 128.8 × 134.0 × 125.1 Å.

The equilibrated protein system was merged with the equilibrated mica layers into a single structure using TopoTools such that the lowest position of protein Cα atoms on the C-terminal face was 25 Å above the average z coordinate of the bridging oxygen atoms on the mica surface (henceforth treated as position *z* = 0). Overlapping waters and ions were translated along the +*z* direction by the periodic **c** vector from the last frame of the protein simulation to minimize bad contacts. Finally, 750 Cl^−^ ions were added to the solution to neutralize the system, bringing the solution ion concentrations to ~2.67 M K^+^ and 3.33 M Cl^−^, close to experimental conditions. Solution-exposed K^+^ ions on both faces of the mica slab were converted to the Joung-Cheatham atom type to reflect their occupancy by solution-phase ions, while K^+^ ions on the interior retained their typing as prescribed by INTERFACE. The resulting system was minimized for 100 steps to remove bad contacts then equilibrated for 1 ns in the NPT ensemble, keeping the periodic **a** and **b** vectors constant at the equilibrated mica dimensions of 129.25 Å and 134.45 Å, respectively. All mica atoms were lightly constrained (1 kcal∙mol^−1^ ∙ Å^−2^) to their initial positions to prevent drift of the slab. The protein was restrained (by its Cα atoms) to its initial COM height (*z* = 50.0 Å relative to the mica surface oxygens), *xy* position, and angle of rotation using 100 kcal∙mol^−1^ restraints during initial equilibration. The average **c** vector over the last 50 ps of equilibration (185.5 Å) was then used to define the periodic cell dimensions for all subsequent runs. Mica hydrogen atoms were then released from constraints and the system was equilibrated for a further 2 ns at constant volume. In order to qualitatively determine whether ^C98^RhuA adsorption occurs directly at the surface or above the ionic slipping plane, the former configuration was obtained by pulling the protein 20 Å towards the mica surface over 5 ns using a 100 kcal∙mol^−1^ ∙ Å^−2^ moving restraint, such that the minimum Cα position was at *z* ≈ 5 Å.

The protein was allowed to adopt a preferred binding configuration (height, rotation) by releasing the COM restraints on the protein *z* position and rotation angle. Instead, a flat protein orientation was maintained in an unbiased fashion by restraining the difference in Cα COM z positions of diagonal protein chains (A-C and B-D) at 0 Å using two 100 kcal∙mol^−1^ restraints; this allowed the full protein to move freely while preserving the relative positions of its individual subunits. Existing constraints on the protein *xy* position and mica heavy-atom coordinates were preserved to simplify the adsorption/desorption pathway. Sampling was carried out over 10 ns of equilibration, with the protein initially relaxing away from the surface by *ca*. 3 Å, indicating that water and ions interact more strongly with the surface and prevent direct adsorption of the protein.

### APBS calculations

Continuum electrostatics calculations of the ^C98^RhuA protein (appropriately protonated using PROPKA 3.1^62^) were carried out using APBS 1.5^63^ to reflect experimental solution self-assembly conditions (pH 7.5, [ions] = 20 mM, *ε*_solvent_ = 78, *ε*_protein_ = 2).

### Equilibration of 3D periodic crystals

Open-state 2 × 2 ^C98^RhuA crystals were constructed as previously described^23^ for both *p*4 and *p*42_1_2 symmetries. These crystals were then crosslinked to their periodic images via disulfide bonds, solvated in a 200.0 × 200.0 × 114.0 Å box (120,609 and 120,531 waters), and neutralized with Na^+^ ions (Joung-Cheatham). Initial coordinates were first minimized for 2000 steps with all protein atoms held fixed, followed by another 1000 step unrestrained minimization. The systems were then equilibrated for 5 ns (followed by 2 ns of production sampling) at constant pressure, keeping the *xy*-dimensions of the box held constant (final equilibrated periodic **c** dimension ≈ 104.5 Å). 100 kcal∙mol^−1^ · protein^−1^ restraints were employed to maintain the difference in Cα COM xy and z positions of diagonally opposed proteins at 0 Å, preserving the structure of the crystal in a minimally biased fashion. The coordinates for the open-state 2×2 *p*4 crystal (extracted after 1 ns of equilibration) were manipulated to generate all other *p*4 lattice conformations for the piezoelectricity simulations via rigid-body rotations/translations of the individual protein units to their idealized dimensions (while preserving the periodic bonding topology). Each conformation was solvated in its own periodic water box of dimensions *a* × *b* × 114.0 Å (where *a* = *b* = the *xy* unit cell dimensions of that conformation) to maintain the periodic disulfide bonding within the crystal plane and equilibrated at constant pressure as described above. Analogous simulations were carried out for ^F88/C98^RhuA (final periodic box size: 182.5 × 182.5 × 124.8 Å). The average protein coordinates over the last 1 ns of simulation served as the model structures for all simulated tapping calculations.

### Calculation of the ^C98^RhuA macrodipole moment

The dipole moment of ^C98^RhuA was calculated from equilibrated protein structures using the VMD “measure dipole” command and visualized using the dipole watcher plugin, both of which yielded values of *ca*. 1200 D. Hydrogens and Zn ions were excluded. We verified the magnitude of the vector using the Protein Dipole Moments Server^10^ using PDB IDs: 1OJR, which yielded a value of 310 D per protein subunit (1240 D total) and 1GT7 (1134 D, full tetramer). We used 1200 D for all dipole potential energy calculations as a representative (and reflective of the equilibrated protein structure) estimate.

### Electret/piezoelectric simulations

The last frame of the equilibrated *p*4 and *p*42_1_2 3D periodic crystal simulations were used as starting coordinates for slab-geometry electret simulations. The protein crystals were recentered such that their disulfides lay at *z* = 0, and solvent/ions were translated across periodic **c** vector until there were equal numbers of water molecules on each side (within tolerance of <0.1% difference). Ions were added to each side of the disulfides to concentrations of 200 mM NaCl such that there were equal numbers of each type of ion on both sides. Systems with neutral net charge and without neutralizing ions (−64 *e*) were constructed to test the effect of system neutrality on membrane potential (none observed). Protein Cα atoms were constrained to their starting z position using a 100 kcal∙mol^−1^ restraint to maintain an equal number of waters on each side over the course of the simulations. Equilibration was carried out for 10 ns using constant-area 2D periodic boundaries (*a* = *b* = 200.00 Å for *p*42_1_2 lattices, and spanning 151.19–200.00 Å for different *p*4 lattice conformations; see Supplementary Table 2 for details), with full-system electrostatics calculated using the Multiscale Summation Method^64^ in NAMD 2.13, which is compatible with both 2D periodic boundaries as well as non-neutral systems. This permitted the calculation of membrane properties using a single crystal by avoiding the problem of ion diffusion back across the periodic boundary, which otherwise would require a double membrane system consisting of >900,000 atoms. Gentle boundary conditions (1 kcal∙mol^−1^ potentials) were enforced above and below the liquid phase to prevent the evaporation of water molecules using the TclBC module of NAMD. Simulations were carried out for 10 ns to ensure full equilibration.

### Disulfide dihedral energy calculations

Disulfide dihedral angles (as depicted in Supplementary Fig. 2) for the nonperiodic disulfide bonds of *p*4 and *p*42_1_2 crystals were extracted from the 2 ns (2000 coordinates) of production sampling from the 3D periodic crystal simulations. The potential energy associated with each disulfide bond conformation was calculated according to the empirical formula described in ref. ^65^.

### Numerical ^C98^RhuA electrochemical potential calculations

To directly calculate the steady-state voltage drop arising from ion segregation across RhuA crystals, volumetric electrostatic potential maps (exclusively due to ions) were computed for all 2D electret simulations using the PMEpot^66^ plugin in VMD for all 10 ns of sampling (10,000 configurations), split into 100-frame blocks. The maps were calculated over *a* × *b* × 200 Å volumes (*a* = *b* = 151.19–200.00 Å depending on lattice conformation) at 1 Å intervals, incorporating the influence of periodic images in the xy dimensions, while remaining pseudo-non-periodic in the *z* dimension by providing an appx. 100 Å air gap between periodic images. An Ewald factor of 0.25 Å^−1^ was used to smooth the charge potentials. The linear voltage drop across the crystal was quantified by integrating over the xy voxels within each 3D volume and projecting the average potential onto the z dimension using a Python script. The difference in electrostatic potential due to the ion distribution across the membrane was calculated, conservatively scaled by the dielectric of pure water (*ε* = 78), and multiplied by the factor 25.87 mV ∙ (*k*_B_*T)*^−1^ for a 300 K simulation. The 5–10 ns time-averaged membrane potentials for *p*4 and *p*42_1_2 crystals were −56.09 ± 5.71 mV and −3.09 ± 5.85 mV, respectively. This potential for p4 crystals is in very close agreement with the potential calculated analytically (−57.96 mV) for open-state *p*4 crystals based purely on the density of dipoles within the lattice (see Supplementary Discussion), strongly suggesting that the macrodipole moment of RhuA crystals can indeed account for their predicted bulk properties.

### Pairwise ^C98^RhuA nanoparticle electrostatic potentials

The electrostatic potential between individual ^C98^RhuA proteins was computed using the pairwise potential between two charged nanoparticles (*U*_*ij*_) proposed by Phillies^29^ for dilute solutions of proteins and charged colloids (Eq. 1), as employed previously for modelling nanoparticle and protein interactions^26,28,67^.

1 $$U_{ij}\left( {r_{ij},\theta _{i,j},\varphi _{i,j}} \right) = \, 	k_e\frac{{q_iq_j}}{{r_{ij}}}e^{ - \kappa r_{ij}}C_0^2 + k_e\frac{{q_i\mu _j\cos \theta _j + q_j\mu _i\cos \theta _i}}{{r_{ij}^2}}e^{ - \kappa r_{ij}}C_0C_1 \\ 	+ k_e\frac{{\mu _i\mu _j}}{{r_{ij}^3}}\left\{ {\cos \theta _j\cos \theta _j\left[ {2 + \kappa r_{ij} + (\kappa r_{ij})^2} \right] \\ + \sin \theta _i\sin \theta _j\cos (\varphi _i - \varphi _j)\left[ {1 + \kappa r_{ij}} \right]} \right\}e^{ - \kappa r_{ij}}C_1^2,$$

where *C*_0_ and *C*_1_ are given by:

2 $$C_0 = \frac{{e^{\kappa a}}}{{1 + \kappa a}},$$

3 $$C_1 = \frac{{3e^{\kappa a}}}{{2 + 2\kappa a + \left( {\kappa a} \right)^2 + \frac{{\left( {1 + \kappa a} \right)}}{\varepsilon }}}.$$

Here, *q*_*i*_ and *q*_*j*_ are the net charge of each ^C98^RhuA protein (−16*e*), *μ*_*i*_ and *μ*_*j*_ are the dipole moments of each ^C98^RhuA protein (1200 D), *a* the particle radius (chosen to be 4.0 nm, the radius for a circle of equivalent area), and *r*_*ij*_ is the center-center distance between particles. The angles *θ*_*i,j*_ are the dipole-*r*_*ij*_ angle for each protein, while (*φ*_*i*_ − *φ*_*j*_) is the dihedral angle between the two dipoles about the *r*_*ij*_ vector (Δ*φ*_*ij*_). *k*_*e*_ is Coulomb’s constant and given as (4π*ε*_0_*ε*)^−1^; *ε*_0_ the permittivity of vacuum; *ε* the relative solvent permittivity (78); and *κ*^−1^ is the Debye screening length (2.1508 nm, corresponding to a solution of 20 mM ions).

The requirement of lateral disulfide bonding within the 2D plane of ^C98^RhuA crystals enables Eq. 1 to be simplified considerably. It geometrically restricts the dipole moment of each particle pair to be oriented normal to the lattice (so *θ* = 90°), and Δ*φ*_*ij*_ = 0 (*p*4 symmetry) or 180 (*p*42_1_2 symmetry). We can then reduce all sin(*θ*) terms to unity, and nullify all cos(*θ*) terms (comprising the entire charge-dipole term and first half of the dipole-dipole term). This gives us the reduced form of *U*_*ij*_ (Eq. 4):

4 $$U_{ij}\left( {r_{ij},\Delta \varphi _{ij}} \right) = k_e\frac{{q_iq_j}}{{r_{ij}}}e^{ - \kappa r_{ij}}C_0^2 + k_e\frac{{\mu _i\mu _j}}{{r_{ij}^3}}\cos (\Delta \varphi _{ij})\left[ {1 + \kappa r_{ij}} \right]e^{ - \kappa r_{ij}}C_1^2.$$

We can see that the first term is purely repulsive due to the identical net charge on both ^C98^RhuA proteins, while the dipolar interaction is dependent on the pair dihedral angle, making it repulsive for *p*4 crystals (cos(0) = 1) but attractive for *p*42_1_2 crystals (cos(180) = −1). The dihedral angle can also be allowed to vary continuously (*i.e*., accessible to a newly-attached monomer to the growing lattice edge), and results in an energy funnel towards the minimum energy configuration of antiparallel packing (Supplementary Fig. 3). This illustrates the origin of the thermodynamic selectivity for *p*42_1_2 lattices in solution (Supplementary Fig. 4).

### Reporting summary

Further information on research design is available in the Nature Research Reporting Summary linked to this article.

## Supplementary information

Supplementary Information

Supplementary Software 1

Reporting Summary

## Data Availability

Data supporting the findings of this manuscript are available from the corresponding authors upon reasonable request. A reporting summary for this article is available as a supplementary information file.

## Code availability

The VMD script (v1.0.1) capable of performing the AFM tapping simulations (and associated 10.5281/zenodo.3924893helper utilities and example files) is available online from R.G.A. via GitHub repository (10.5281/zenodo.3924893) under a GNU General Public License v3.0.
