# Supplementary material for: Assembly of a patchy protein into variable 2D lattices via tunable multiscale interactions
Source: Nat Commun. 2020 Jul 28;11:3770. doi: 10.1038/s41467-020-17562-1 (PMC7387446; doi:10.1038/s41467-020-17562-1)
Supplement: Supplementary file 1 — Supplementary Information [file 41467_2020_17562_MOESM1_ESM.pdf]

SUPPLEMENTARY INFORMATION

Assembly of a patchy protein into variable 2D lattices via tunable multiscale interactions

Shuai Zhang[1,2,†], *Robert G. Alberstein*[3,†], *James J. De Yoreo*[1,2,*], *F. Akif Tezcan*[3,4,*]

[1]Department of Materials Science and Engineering, University of Washington, Seattle, WA 98195, USA.

[2]Physical Sciences Division, Pacific Northwest National Laboratory, Richland, WA 99352, USA.

[3]Department of Chemistry and Biochemistry, University of California, San Diego, La Jolla, CA 92093, USA.

[4]Materials Science and Engineering, University of California, San Diego, La Jolla, CA 92093, USA.

*Correspondence to: tezcan@ucsd.edu (F.A.T.); james.deyoreo@pnnl.gov (J.J.D.Y.).

†These authors contributed equally to this work.

This Supplementary Information file includes:

Supplementary Figures 1-16………………………………………………… S2 – S17
Supplementary Tables 1-2………………………………………………… S18 – S19
Supplementary Discussion………………………………………………… S20 – S25
Supplementary References………………………………………………… S26 – S26

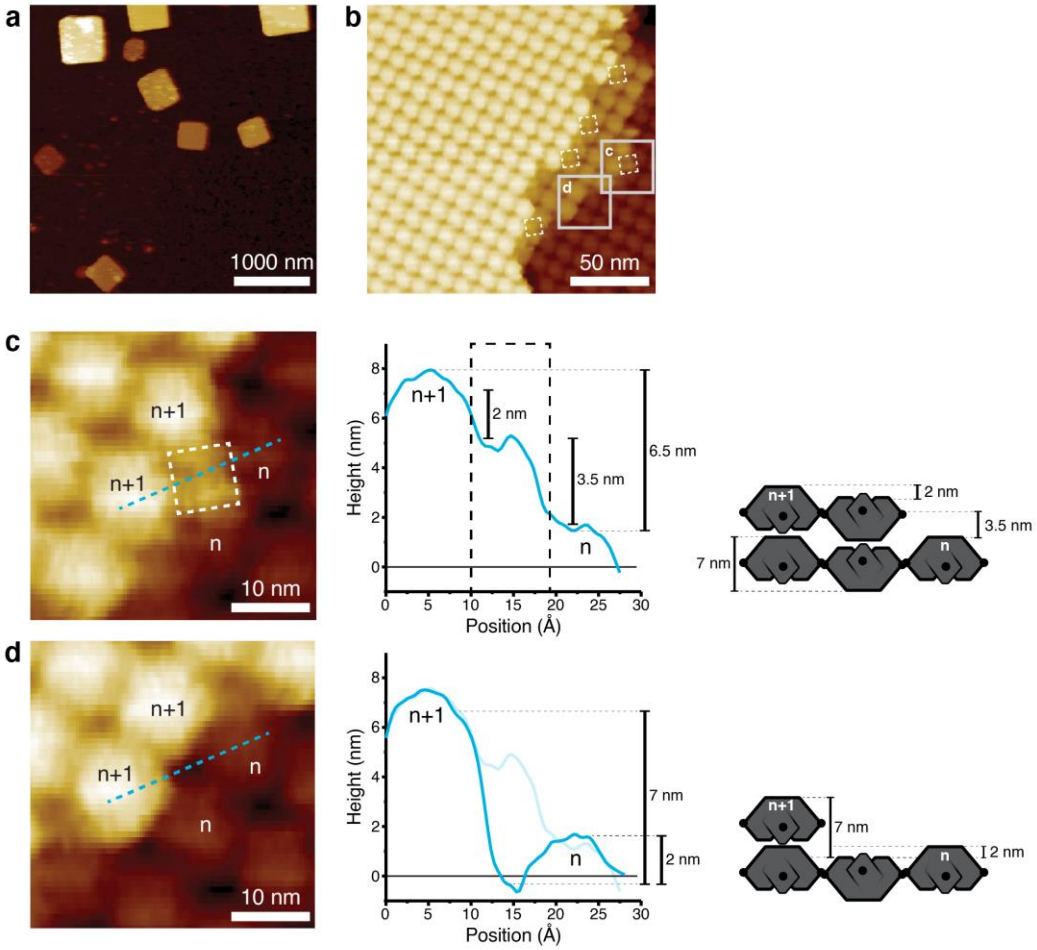

Supplementary Figure 1 | Step-edges of $p42_12$ crystals. a, Low-magnification image of stacked solution-grown $p42_12$ crystals deposited onto poly-lysine covered *m*-mica. **b,** High-magnification image of the crystal edges, showing the stacking of layers. Dangling Nterm-up proteins are highlighted with white dashed boxes in **b** and **c**. The solid light gray boxes in **b** indicate regions selected for more detailed analysis (**c** and **d**). **c,** Close-up AFM image of a terminal Nterm-up protein with corresponding height trace and cartoon to highlight the stepped geometry of the crystal edge. **d,** Same as c but without a terminal Nterm-up protein, showing the expected *ca.* 7 nm drop in height for two stacked $p42_12$ crystals. The height trace from **c**, shifted by *ca.* −0.374 nm to align its starting height relative to **d** at 0 Å position, is reproduced faintly to facilitate comparison. Height ranges: 140 nm (**a**), 35 nm (**b**), 12 nm (**c**/**d**).

26

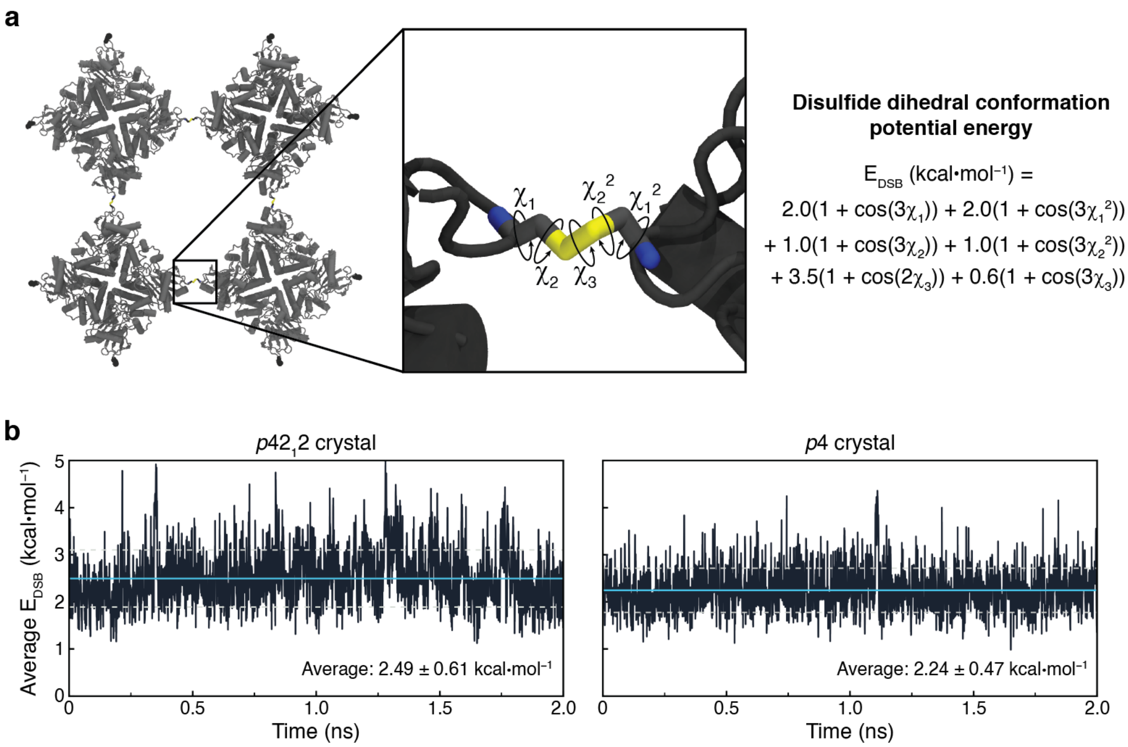

Supplementary Figure 2 | Lack of conformational preference of disulfide bonds. a, Close-up rendering of one of the four disulfide bonds not connected through the periodic boundaries in the 2×2 structure. The five dihedral angles which fully define a disulfide conformation are labeled in the inset, with the equation which relates them to their dihedral potential energy[12] on the right. **b,** Plots of the average dihedral potential energy for all four non-periodic disulfides over the final 2 ns of sampling from the 3D 2×2 simulations. The time average is marked with a cyan line and boundaries of ±1 standard deviation are delineated with dashed light grey lines. As the average energies are within error of each other, the atomic configurations of the disulfide bonds do not appear to impart any energetic preference towards one symmetry vs the other. These disulfide bonds are the only true connection point between RhuA protein units, and the protein interfaces are too far apart to form traditional H-bonds, salt bridges, or hydrophobic contacts, so the lack of any energetic bias here indicates that any forces responsible for dictating the particular symmetry of RhuA lattices must be a long-range potential acting at several-nm distances.

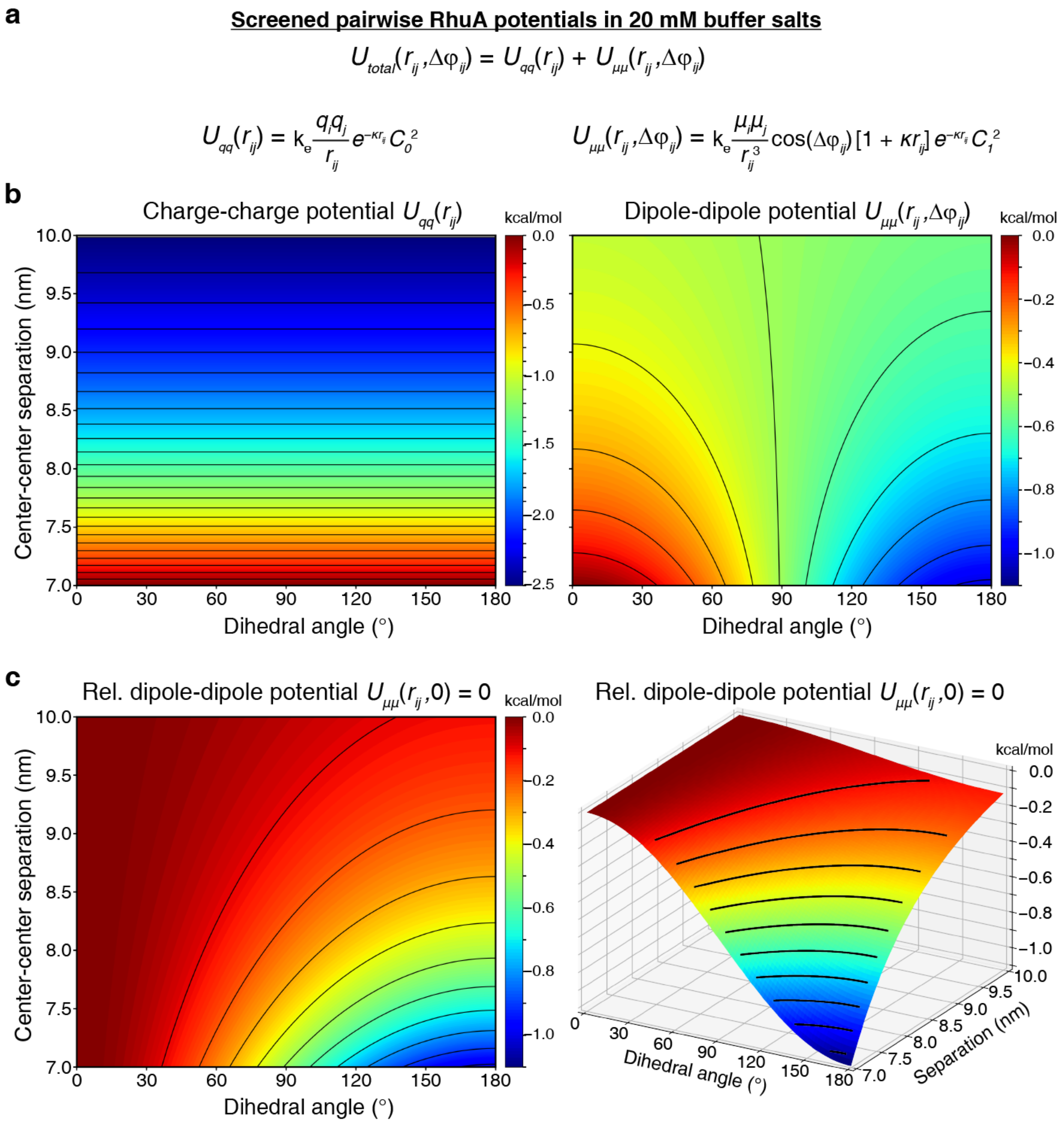

$$U_{total}(r_{ij},\Delta\varphi_{ij}) = U_{qq}(r_{ij}) + U_{\mu\mu}(r_{ij},\Delta\varphi_{ij})$$

$$U_{qq}(r_{ij}) = k_e \frac{q_i q_j}{r_{ij}} e^{-\kappa r_{ij}} C_0^2 \qquad U_{\mu\mu}(r_{ij},\Delta\varphi_{ij}) = k_e \frac{\mu_i \mu_j}{r_{ij}^3} \cos(\Delta\varphi_{ij}) [1 + \kappa r_{ij}] e^{-\kappa r_{ij}} C_1^2$$

Supplementary Figure 3 | ^{C98}RhuA nanoparticle pairwise electrostatic potential energy surfaces. a, Reduced form of the electrostatic nanoparticle-nanoparticle interaction potential (Equation 4, Methods) and its individual components (charge and dipolar). **b,** Absolute potential energy landscapes for each component of the total potential, shifted such that the maximum energy is at 0.0 kcal/mol, highlighting the dihedral dependence unique to the dipole-dipole potential. The global maximum and minimum energies correspond to $\Delta\varphi_{ij}$ = 0 (parallel dipoles) and $\Delta\varphi_{ij}$ = 180 (antiparallel dipoles), respectively, at 7 nm separation. **c,** 2D and 3D representations of a relative dipole-dipole potential, shifted such that the energy at $\Delta\varphi_{ij}$ = 0 (parallel dipoles) is set to 0.0 kcal·mol^{-1} for all distances, revealing the potential energy funnel created by dipolar interactions over all r_{ij}. Black contour lines in all plots represent decrements of 0.1 kcal·mol^{-1} relative to 0.0.

28

a

Pairwise nanoparticle dipole-dipole potential

$$U_{\mu\mu}(r_{ij}) = \frac{\mu_i \mu_j}{4\pi\varepsilon\varepsilon_0 r_{ij}^3}\cos(\Delta\varphi_{ij})[1 + \kappa r_{ij}]e^{-\kappa r_{ij}} C_1^{\,2}$$

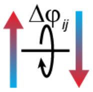

Lattice-incorporated monomer energy

$$\mu_{RhuA}(monomer) = \sum_j^N U_{\mu\mu}(monomer, j)$$

b

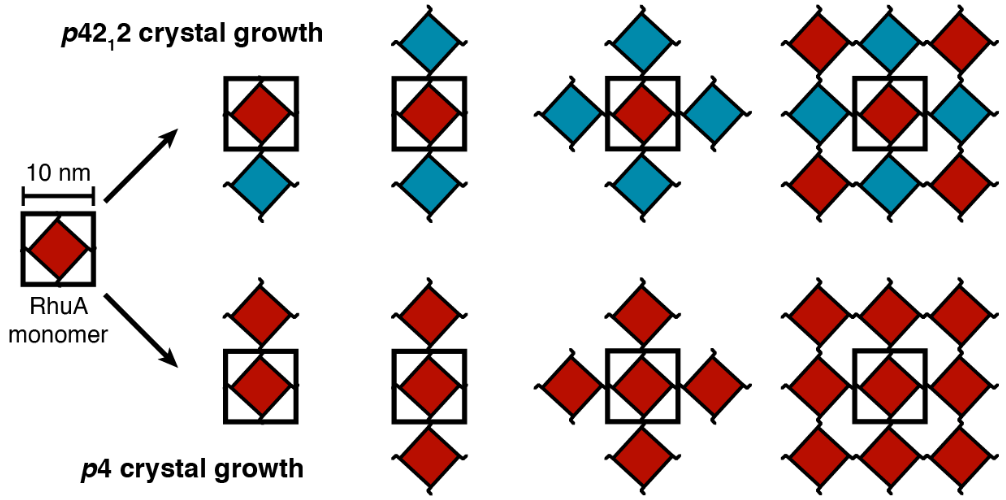

**# neighbors**	**N = 1**	**N = 2**	**N = 4**	**N = 8**	
$\mu_{RhuA}(p42_12)$ =	−0.063	−0.126	−0.251	−0.234	(kcal•mol⁻¹)
$\mu_{RhuA}(p4)$ =	+0.063	+0.126	+0.251	+0.268	(kcal•mol⁻¹)
$\Delta\mu_{RhuA}$(open) =	−0.126	−0.251	−0.502	−0.502	(kcal•mol⁻¹)
$\Delta\mu_{RhuA}$(closed) =	−1.052	−2.104	−4.208	−4.208	(kcal•mol⁻¹)

Supplementary Figure 4 | Nearest-neighbor amplification of dipole-dipole interactions. a, Reduced form of the dipole-dipole interaction potential (Equation 4, Methods) and its extension to calculate the total dipolar energy felt by a single monomer as it is incorporated into a lattice under experimental self-assembly conditions (aqueous 20 mM buffer solution). **b,** Cartoon depicting the nucleation of both *p*4 and $p42_12$ crystals around a central ^{C98}RhuA monomer (**top**) and its cumulative dipole potential energy (μ_{RhuA}) as a function of crystal symmetry and number of nearest neighbors (**bottom**). Individual energies for all sizes are included for the open-state crystals (10 nm protein separations), while the potential energy difference ($\Delta\mu_{RhuA}$) is also reported for the closed state (7.071 nm protein separations) to depict the range of magnitudes which promotes antiparallel packing during solution self-assembly. Attenuated energies at the growing edge facilitate reorientation of newly attached monomers before being "locked in" by nearest-neighbor interactions upon incorporation into the bulk lattice. The total potential converges within one shell of neighbors, as shown by the negligible change in $\Delta\mu_{RhuA}$ for $N \geq 4$.

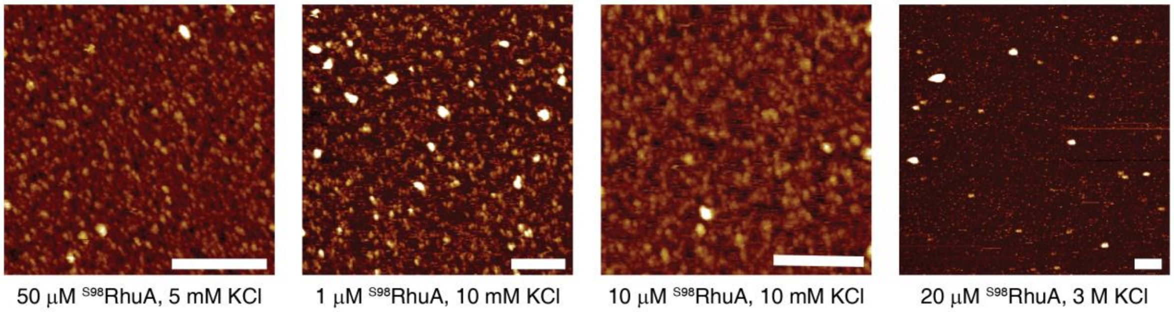

Supplementary Figure 5 | Attempted self-assembly of ^{S98}RhuA on *m*-mica. AFM images of ^{S98}RhuA incubated on *m*-mica at various concentrations of protein and KCl. Crystallization did not occur under any conditions explored here. Scale bars: 200 nm. Height range: 16 nm.

30

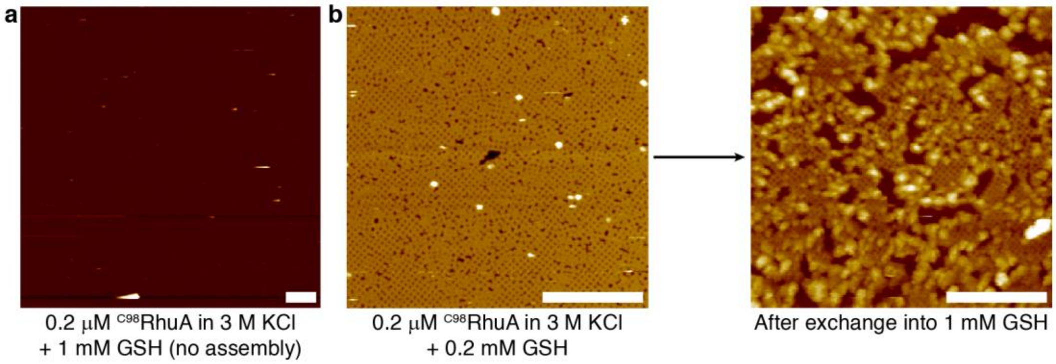

Supplementary Figure 6 | Effect of reductant on surface-grown lattices. a, No self-assembly was observed after a 48h incubation on *m*-mica in the presence of 1 mM reduced glutathione (GSH). **b,** Surface-grown crystalline networks (formed after 48h incubation on *m*-mica) undergo disassembly upon the introduction of additional GSH, demonstrating that disulfide bond crosslinks are essential to maintain the protein lattice structure. Scale bars: 200 nm. Height range: 8 nm.

31

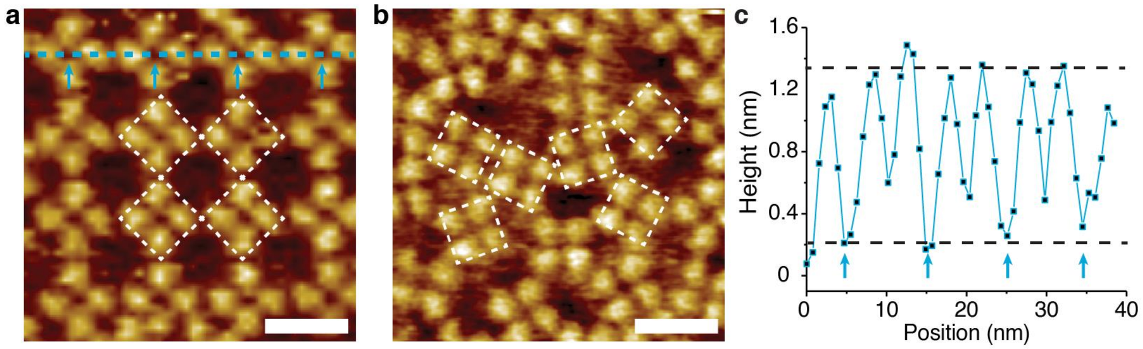

Supplementary Figure 7 | High-resolution images of Nterm-up ^{C98}RhuA proteins. AFM images of Nterm-up ^{C98}RhuA crystals (**a**) and disordered monomers (**b**). White boxes indicate representative individual proteins. **c**, Height profile along the line trace in **a**. The blue arrows in **a** and **c** denote the central dip of the protein N-terminus. Scale bars: 10 nm. Height range: 3 nm.

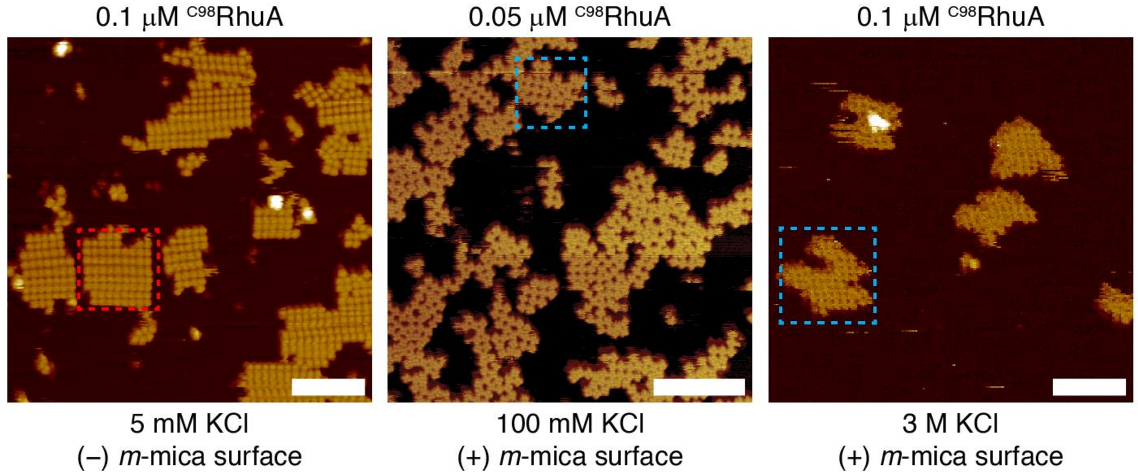

Supplementary Figure 8 | Absolute crystal orientation selectivity reflects mica surface charge. Self-assembly of very low [^{C98}RhuA] onto *m*-mica yields small isolated crystalline domains of identical orientation, likely corresponding to regions of (higher) homogenous local surface charge. At 5 mM KCl, the heterogeneous distribution of K^+ ions is insufficient to prevent preferential binding of the cationic ^{C98}RhuA N-terminus to the negative surface. This is in contrast to the observed heterogeneity when the surface is fully covered by protein (**Fig. 2a**). Assembly in ≥100 mM KCl exclusively yields open-state Nterm-up crystals, as observed for higher protein concentrations. Red and blue boxes highlight representative Cterm-up and Nterm-up crystals, respectively. Scale bars: 100 nm. Height range: 12 nm.

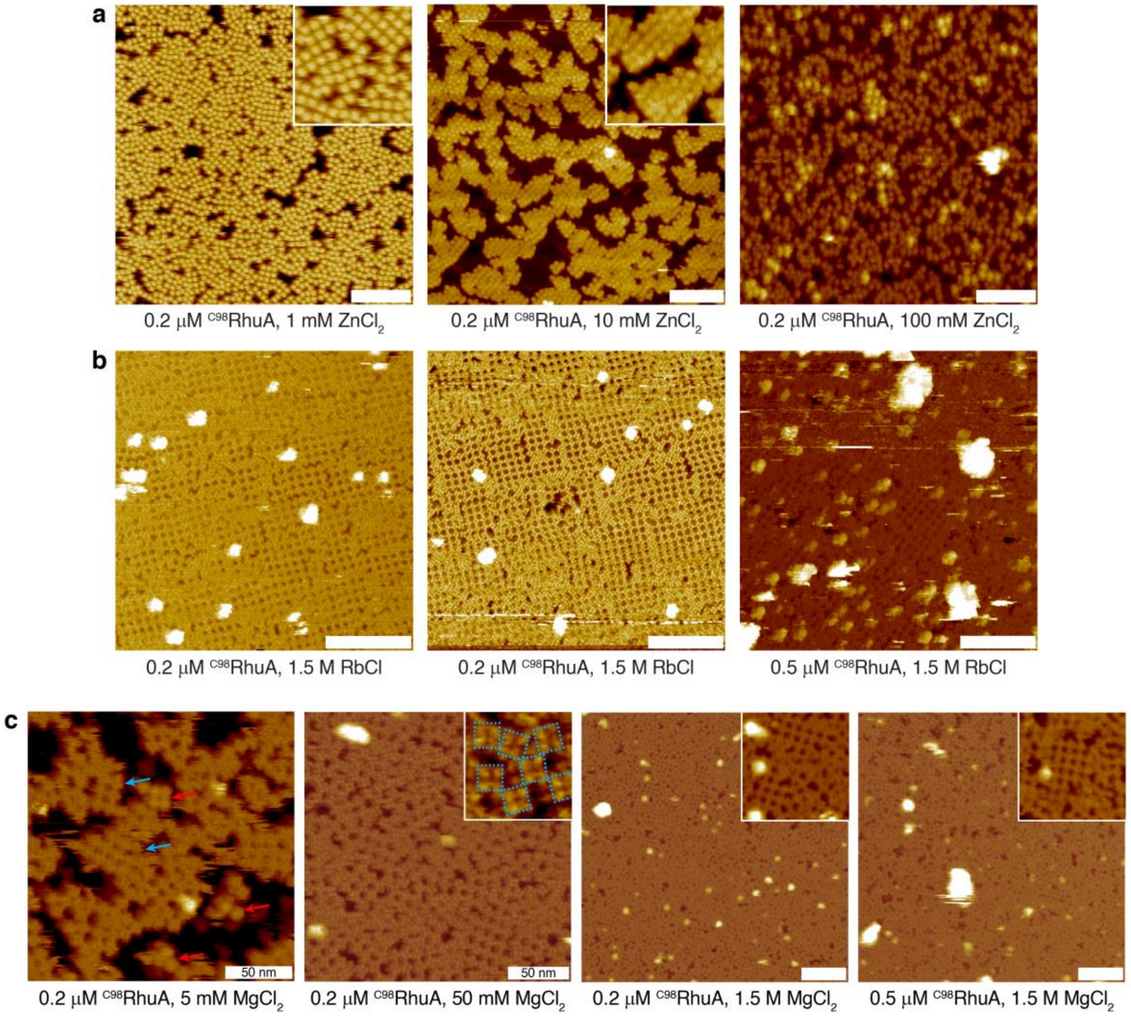

Supplementary Figure 9 | Specific ion effects on ^{C98}RhuA self-assembly on *m*-mica. Mica-templated self-assembly of ^{C98}RhuA at low concentrations in the presence of **a,** Zn^{2+}, **b,** Rb^{+}, and **c,** Mg^{2+}. Zn^{2+} ions do not effectively bind the surface, so it remains negative and enforces exclusively Cterm-up binding, regardless of [Zn^{2+}]. Rb^{+} ions are known substitutes for K^{+} ions in the surface vacancies, and thus yield analogous Nterm-up crystals at molar concentrations. **c**, Increasing concentrations of Mg^{2+} ions recapitulates the trend in bound protein/crystal orientation observed for K^{+} (and Rb^{+}), but reversal occurs at half of the monovalent concentration, suggesting that all three ions modulate the mica surface charge upon adsorption. Red and blue arrows/boxes identify Cterm-up and Nterm-up oriented proteins, respectively. Scale bars are 100 nm except where noted. Height ranges: 8 nm (**a**/**b**), 12 nm (**c**).

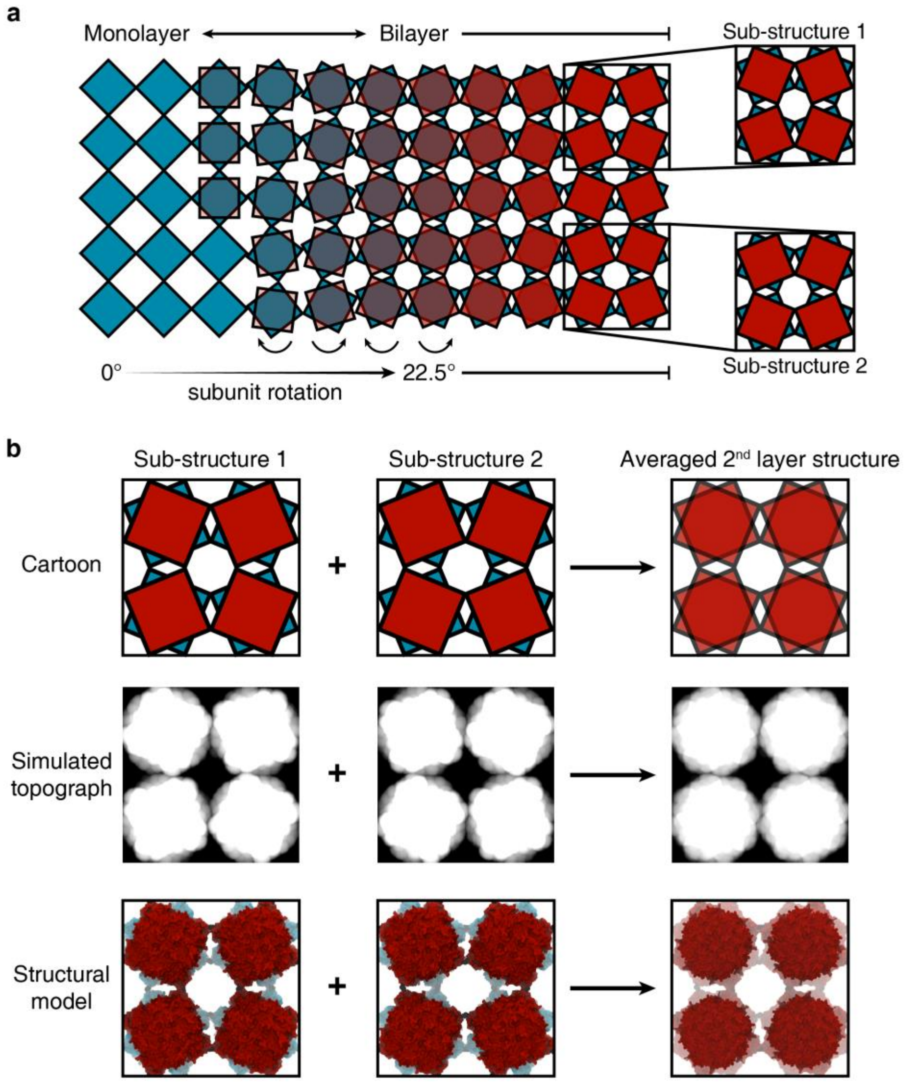

Supplementary Figure 10 | Validation of the ^{C98}RhuA bilayer crystal morphology via tapping simulations. a, Expanded cartoon from **Fig. 3h**, highlighting the requisite 22.5° rotation of the first layer to bring 2nd layer proteins into disulfide-bonding distance (visible underneath low-opacity 2nd layer proteins on left) and resultant "sub-structures" arising from this geometric constraint. **b,** Depiction of the contribution of each sub-structure to the final average topograph, which is consistent with the experimental correlation average for the bilayer crystals.

35

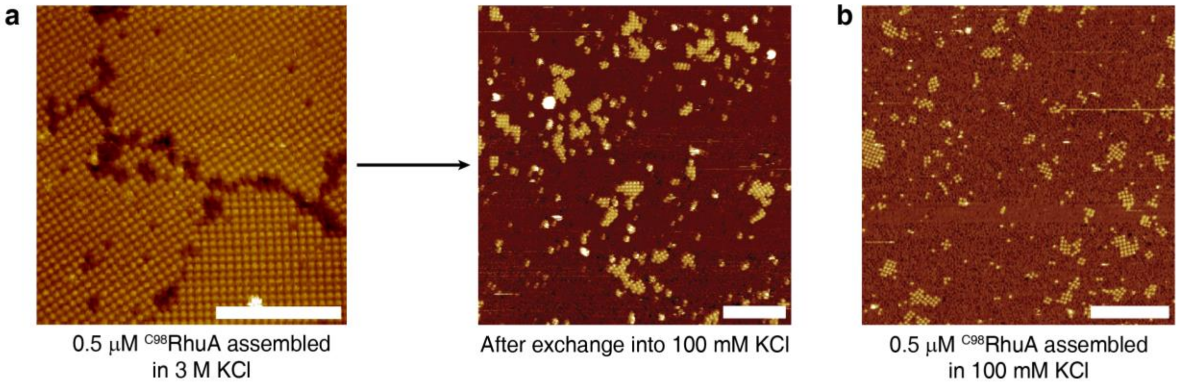

Supplementary Figure 11 | Exfoliation of bilayer crystals. a, AFM images of ^{C98}RhuA bilayer crystals grown in the presence of 3 M KCl before (left) and after (right) exchanging buffer with 100 mM KCl solution. Most of the second layer falls away from the underlying monolayer due to loss of the salting-out effect from the high [KCl]. In this state these crystals resemble ^{C98}RhuA crystals grown directly in the presence of 100 mM KCl (**b**). Scale bars are 200 nm. Height range: 12 nm.

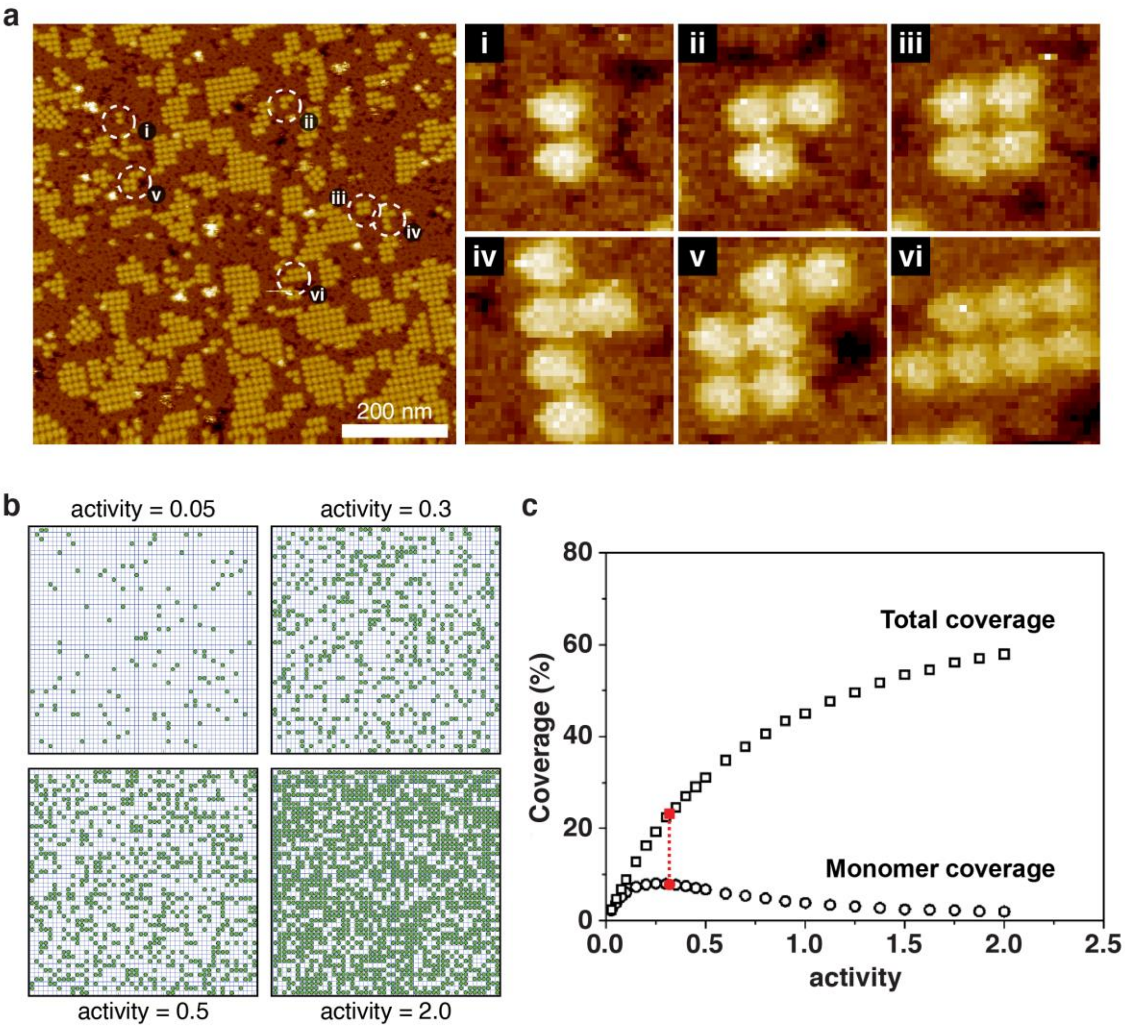

Supplementary Figure 12 | Rarity of isolated 2nd-layer monomers in kinetically-trapped assemblies. a, AFM image of 5 µM ^{C98}RhuA assembled in the presence of 3 M KCl. Large regions of the first protein layer are non-crystalline due to the high concentration of protein, which precludes the formation of large bilayer domains. Only 0.35% of all 2nd layer proteins exist as monomers isolated from any nearest neighbors. Instead, nearly all 2nd layer domains consist of at least two neighboring monomers (**i-vi**; labeled regions and zoom-in images), suggesting that disulfide bonds are essential for the stability of bilayer crystals. The image on the left is the uncropped version of **Fig. 3g**. Height ranges: 12 nm (large scan area, left), 8 nm (high-magnification images, right). **b, c**, Relationship between total adsorbate coverage and corresponding isolated monomer coverage determined by grand canonical Monte Carlo simulation for random adsorption, desorption and displacement of non-interacting species with differing solution activities onto a 2D square lattice (adapted with permission from supplementary ref. 13. Copyright 2018, American Chemical Society). **b** shows the spatial distribution of adsorbates and **c** shows the equilibrium adsorbate coverage, as well as the coverage of isolated monomers. The dashed line marks the total coverage of 23% obtained at 5 µM ^{C98}RhuA and 3 M KCl and the corresponding expected monomer coverage of 7% for non-interacting particles. Were these particles (2nd layer monomers) subject to repulsive interactions, this preference would manifest as a higher percentage of isolated monomers across all total coverage values. The low number of isolated 2nd layer monomers observed in our work (0.35%) thus indicates strongly favorable inter-monomer interactions (disulfide bonding) which are essential for stability of the 2nd layer.

37

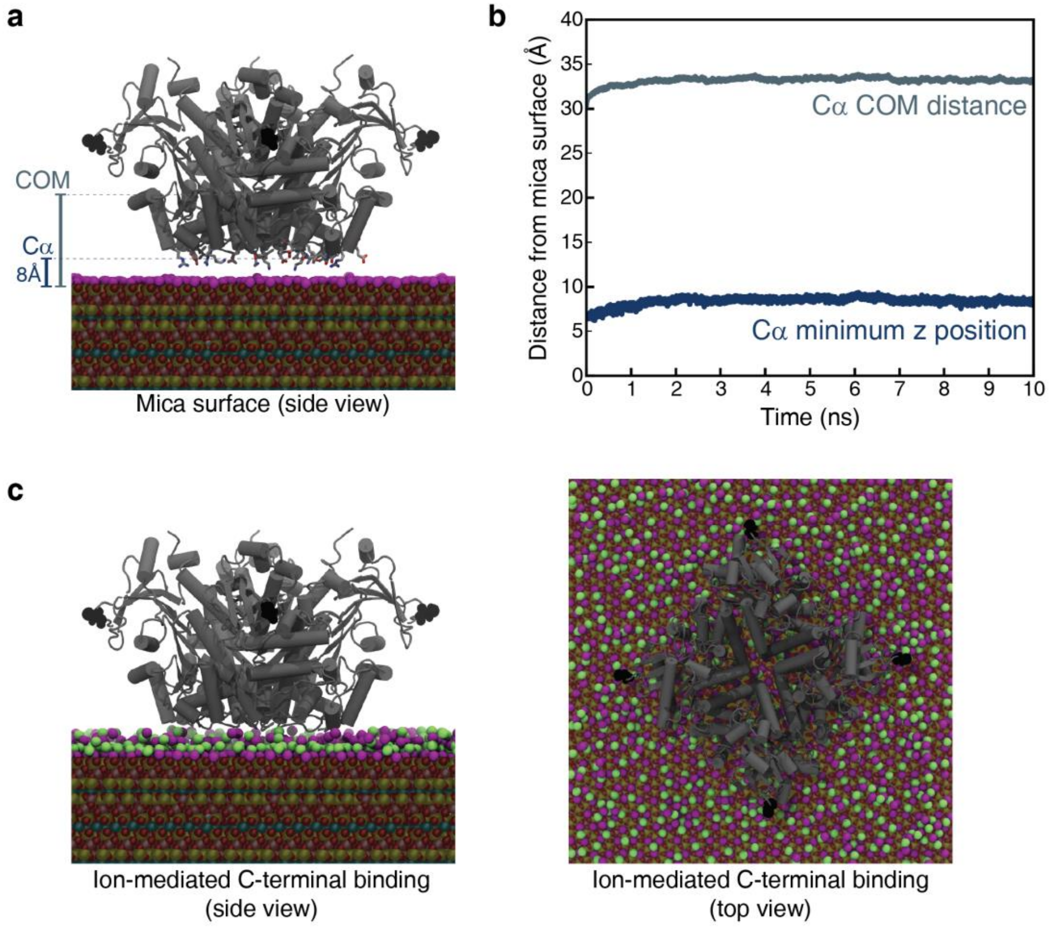

Supplementary Figure 13 | Preferred binding geometry of RhuA C-terminus to *m*-mica in 3 M KCl. a, Rendering of ^{C98}RhuA associated with the *m*-mica surface at its equilibrium position (8–9 Å away from the surface). All residues with any atom ≤7 Å away are shown as sticks. The COM and Cα distances are depicted on the left. **b,** Plot of the change in protein-mica distance over 10 ns of equilibration; the protein reaches its equilibrium distance within 2–3 ns. **c,** Side and top views of **a** with all K^+ and Cl^- ions within 7 Å of the surface depicted as purple and green spheres, respectively. The observed double-layer ion behavior is typical of a charged surface, and mediates all protein-surface interactions, enabling the facile movement of ^{C98}RhuA on the surface by avoiding direct contacts. All renders are from the same snapshot at 5 ns of equilibration.

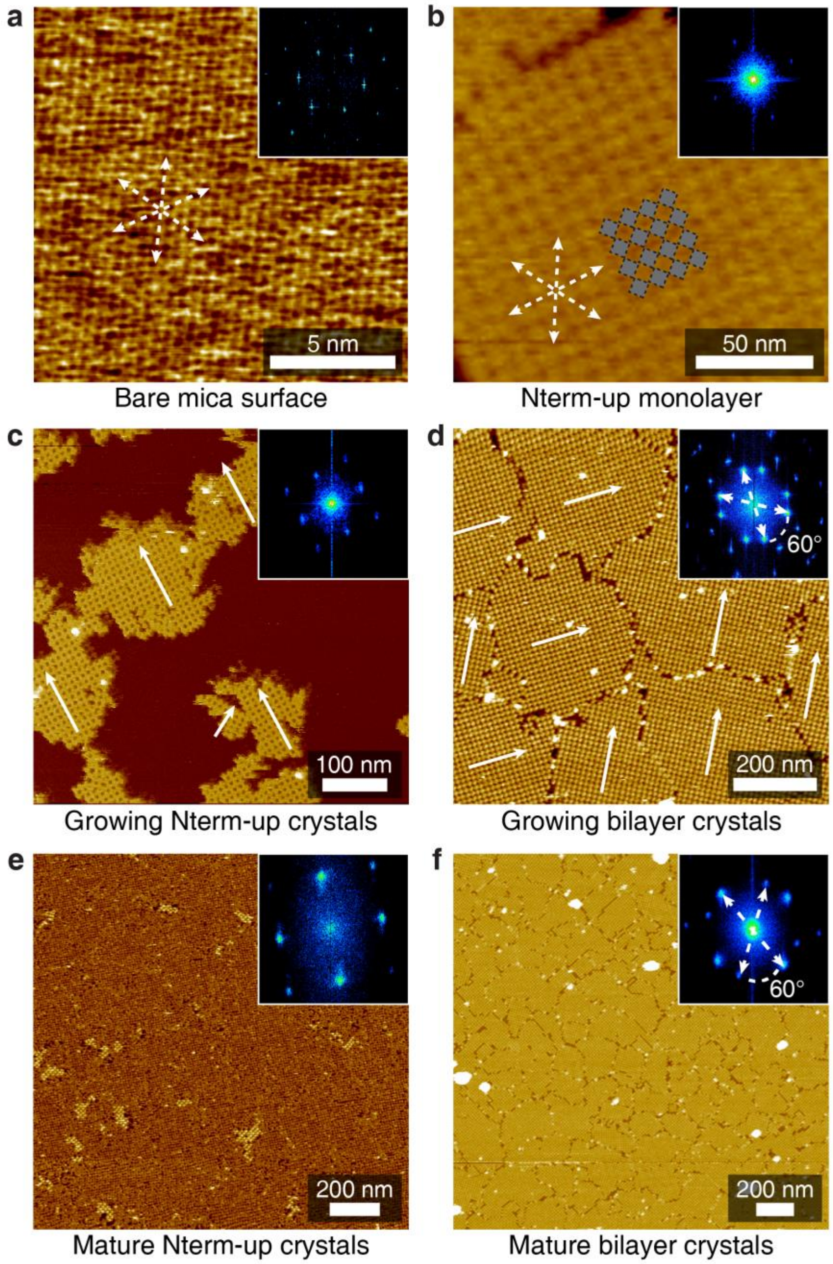

Supplementary Figure 14 | Domain alignment along mica vectors. a, AFM image of the bare mica surface. The hexagonal arrangement of the mica lattice can be directly visualized, and the corresponding lattice vectors are drawn as dashed arrows. **b,** Nterm-up monolayer crystals imaged with the same underlying mica orientation, showing the alignment of ^{C98}RhuA proteins relative to the mica lattice vectors. **c, d,** Examples of crystal domain growth along preferred directions (indicated by arrows). **e, f,** Low-magnification images of mature crystals, demonstrating that crystallite domain orientations are preserved over thousands of unit cells. Growth directions are separated by 60°, consistent with the influence of the underlying substrate. The insets are FFTs. Height ranges: 0.2 nm (**a**), 16 nm (**b**), 8 nm (**c**/**d**), 12 nm (**e**), 10 nm (**f**).

39

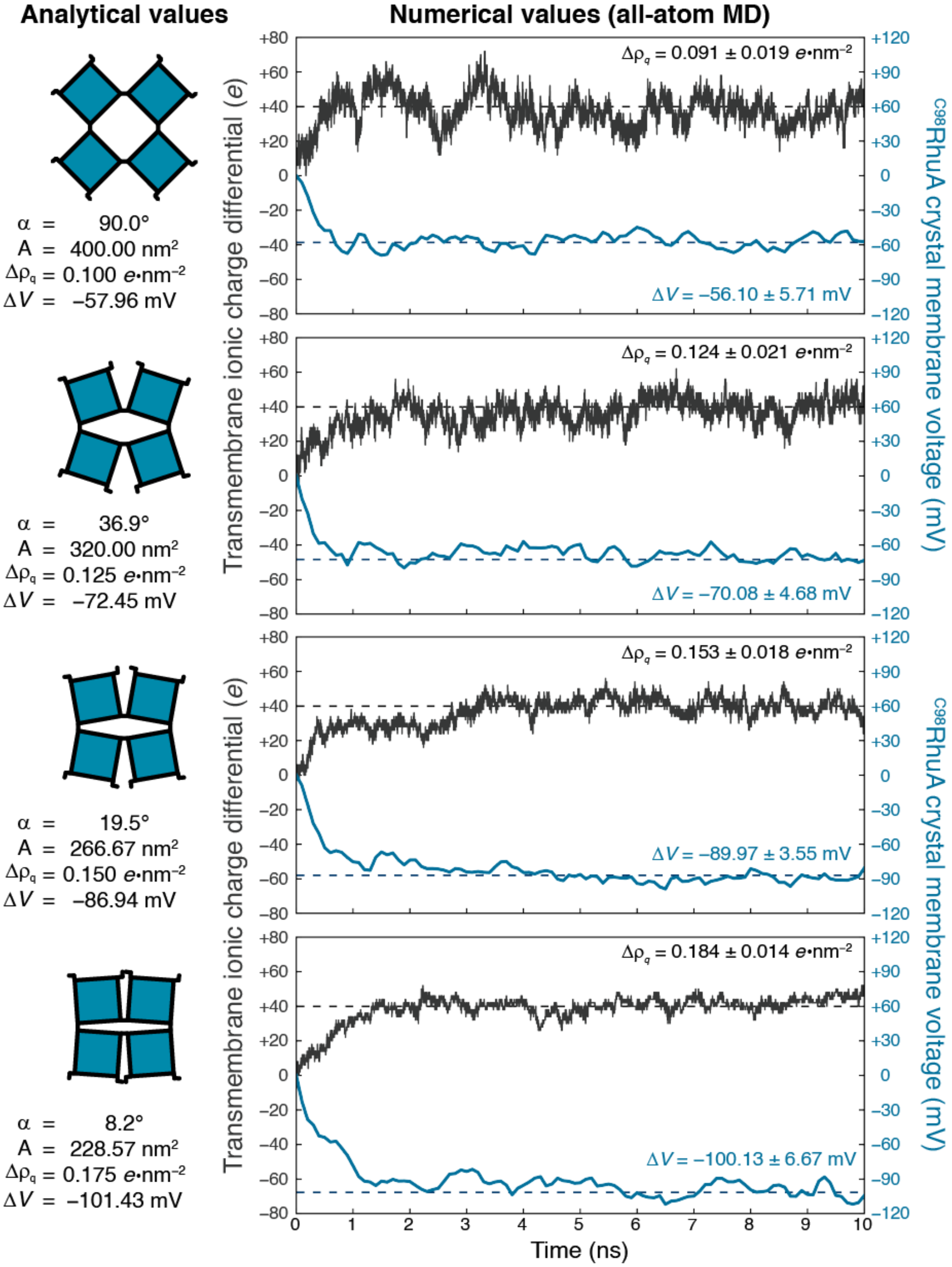

Supplementary Figure 15 | Piezoelectric behavior of *p*4-symmetry ^{C98}RhuA lattices. Results of all-atom MD simulations of *p*4-symmetric ^{C98}RhuA crystals at different conformational states. Cartoons and theoretical values for the hinge angle (α), unit-cell area (A), polarization density (ρ_μ), and electric potential difference (ΔV) are shown at left. The trans-lattice ionic charge differentials (Δq) and resultant voltages (gray and blue traces, respectively) over 10 ns of sampling are shown at right. $\Delta\rho_\mu$ and ΔV values within each plot are the average and s.d. of each quantity over the last 5 ns of simulation. The dashed lines correspond to the idealized analytical value of ΔV for each conformation, and $\Delta q = +40e$ (all conformations).

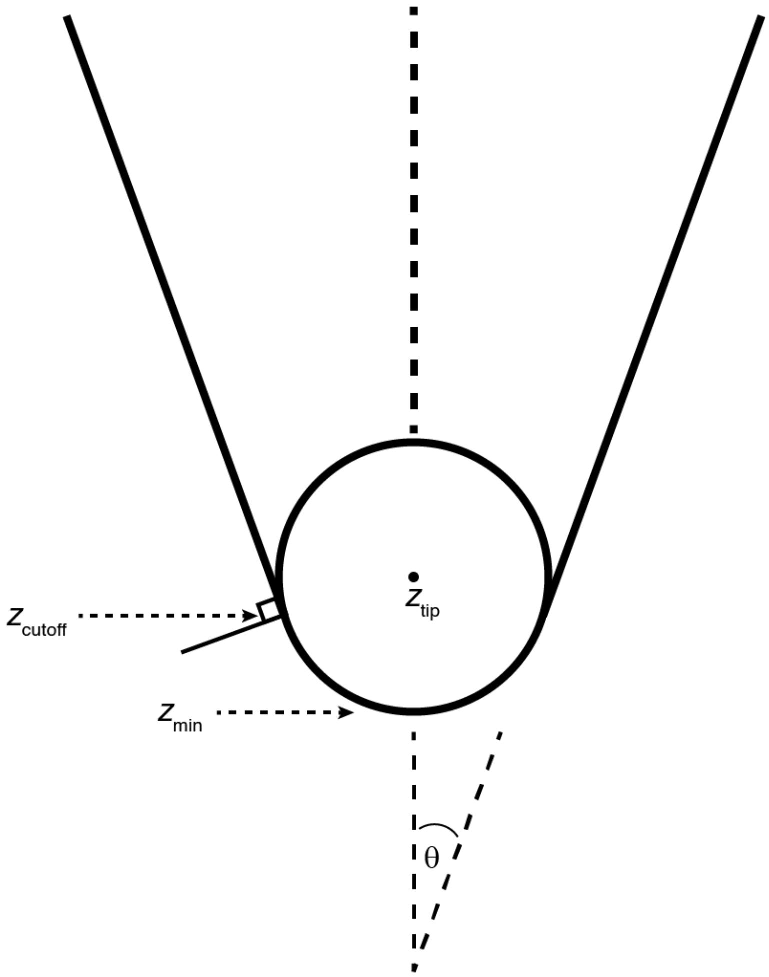

Supplementary Figure 16 | Description of tip convolution simulations. The effect of tip convolution was modeled by looking for overlaps between protein atoms and a tip with the above geometry. Checks were performed by first evaluating the volume encompassed by a sphere centered at z_{tip}, then testing for any overlaps within a cone of half-angle θ which lie tangent to the spherical tip. Upon finding a position at which the number of overlaps met the cutoff criterion, the final height was reported as z_{min}. This effectively captures the coarsening of atomic structure observed during AFM experiments.

no KCl	
Window (Å)	k_f (kcal·mol^{-1}·Å^{-2})
46.5	2.0
48.5	2.0
50.5	2.0
52.5	2.0
54.5	2.0
56.5	2.0
58.5	2.0
60.5	2.0

3 M KCl	
Window (Å)	k_f (kcal·mol^{-1}·Å^{-2})
46.5	2.0
47.5	4.0
48.5	2.0
50.5	2.0
52.5	2.0
53.0	4.0
54.5	2.0
56.5	2.0
58.5	2.0
60.5	2.0

Supplementary Table 1 | Umbrella sampling details. Window centers and force constants used for the calculation of the ^{C98}RhuA dimerization PMFs.

Symmetry	Hinge angle (α)	PBC *xy* dims. (Å)	Appx. *z* dim. (Å)	# Na^+ ions	# Cl^- ions
*p*4	8.2	151.186	101.0	212	212
*p*4	19.5	163.299	102.0	264	264
*p*4	36.9	178.885	103.3	336	336
*p*4	90.0	200.00	104.5	448	448
$p42_12$	90.0	200.00	104.5	448	448

Supplementary Table 2 | Piezoelectric membrane simulation details. Information regarding the symmetry, crystal conformation (as hinge angle), periodic box dimensions ($x = y$ and are fixed values, z fluctuates), and total number of ions for all simulations used for the piezoelectricity response analysis. Protein center-center distances between disulfide-bonded dimers are equal to half of the periodic box *xy* dimensions (*e.g.*, 90° angle disulfide-bonded dimers are separated by 100.00 Å).

Supplementary Discussion

Here we analyze and critically assess our approximation of ^{C98}RhuA proteins as biological nanoparticles possessing internal electric dipoles, which enables the use of rigorous analytical expressions for estimating the magnitude (and thus relative importance) of anisotropic electrostatic interactions in dictating the crystal packing of ^{C98}RhuA monomers within 2D lattices. Though inherently approximate, this approach has been broadly applied with great success to the self-assembly of inorganic nanoparticles[1-3], enabling a quantitative dissection of the energetic drivers which impart morphological selectivity (and diversity), particularly for "patchy" nanoparticles possessing multiple distinct interaction modalities that collectively define the self-assembly free-energy landscape. Indeed, dipole-dipole interactions have been repeatedly identified as the decisive mechanism underpinning the selective formation of 1D chains[2] and antiparallel 2D lattices[1,3] over nonspecific globular aggregates more typical of isotropic particles, and have been exploited to construct crystalline materials with specified physicochemical[4] and electronic properties[5] (*e.g.*, COFs[6], piezoelectrics[7]). Given this extensive precedent for the importance and versatility of dipolar interactions in controlling self-assembly (and emergent materials properties), the present discussion is motivated by the possibility that such effects could be applied to "patchy protein" nanoparticles.

While the influence of dipole moments in proteins has been studied for decades[8-11], these interactions are frequently considered in the context of individual α-helices and have been shown to be relatively localized in space. Consequently, it is essential to establish whether or not the global-scale electric field arising from a protein's folded structure and particular arrangement of charged residues across its surface can be approximated with sufficient accuracy using simple dipoles. It is possible that for many proteins this is not the case, owing to a diffuse distribution of

charges on a typical protein's surface. However, the localization of charged residues primarily to the RhuA protein termini surfaces gives rise to a highly polarized atomic structure (and corresponding electric field; **Fig. 1c**), which is considerably more reminiscent of an electric dipole. Therefore, to validate the use of such an approximation for this system, we compare the predicted electrochemical properties of RhuA crystals—calculated from analytical expressions—directly to results obtained numerically from all-atom MD simulations. As outlined below, we find near-quantitative agreement across all methods. Finally, we note that while the validity of analytical dipole approximations likely holds only for proteins with polarized surfaces, such structures are in principle simple to rationally engineer or design *de novo* into other protein building blocks, facilitating the ability to estimate the magnitude of inter-protein dipolar potentials with reasonable accuracy.

Oriented-dipole electret materials possess a permanent electric field owing to the alignment of polarized units within their structure, and the magnitude of this field will be determined by the magnitude and density of these dipoles. We have reported here trans-lattice ion distributions and calculated electrostatic potential differences across open-state *p*4 and $p42_12$ ^{C98}RhuA crystals (**Fig. 5a**), as well as for multiple conformations of *p*4 crystals (**Fig. 5c**, **Supplemental Fig 15**). These results clearly show that the field is present exclusively for the polar *p*4 crystal symmetry, so we next ask whether or not the results are in agreement with expectations based purely on simple dipoles. By doing so, we can critically evaluate whether the magnitude of such dipole-dipole interactions (as calculated from analytical dipoles) are sufficiently accurate to explain the observed antiparallel packing of solution-grown ^{C98}RhuA crystals.

First, let us compare the observed ion distribution across *p*4 crystals from all-atom simulations to expected values calculated using analytical dipoles. To perhaps the most trivial approximation, we can estimate the trans-lattice ion differential expected to arise from dipolar particles simply by converting the 1200 D ^{C98}RhuA macrodipole moment to an equivalent charge × distance "discrete" dipole (0.02082 $e\cdot\mathrm{nm}\cdot\mathrm{D}^{-1}$) of 24.983 $e\cdot\mathrm{nm}$. Taken to run the 5 nm height of the protein, we obtain our "discrete dipole" approximation of a +5.0*e* and −5.0*e* charge pair separated by a distance of 5 nm. Now applying this to the set of 4 proteins in a 2×2 unit cell which comprise our *in silico* model (**Fig. 5**), we expect an accumulation of four +5*e* charges on one side and four −5.0*e* charges on the other, for a net ionic charge differential (Δq) of 40*e* across the lattice.

Indeed, we find that for all conformations of *p*4 crystals subjected to all-atom MD simulations, Δq asymptotically approaches 40*e* (**Supplementary Figure 15**), indicating that the predicted distribution of counterions simply equalizes the net charge of these "discrete dipoles", which remains the same regardless of crystal conformation (open vs. closed). We observe in our simulations that as the lattices become more compact, this value becomes more exactly correct (**Supplementary Figure 15**), likely correlating with changes in total pore area through which the ions can exchange freely (suppressing fluctuations when more compact). This analysis suggests that the treatment of ^{C98}RhuA proteins as analytical dipoles accurately approximates the bulk-scale materials properties similarly predicted by expensive all-atom numerical simulations, indicating that RhuA's electric field may be well-represented quite generally even by this extremely simple model.

Next, we compare the numerically and analytically determined values for the potential drop across *p*4 lattices. The consequence of maintaining a constant trans-lattice ion differential (*i.e.*, independent of the xy unit cell dimensions) is that the charge density on each side of the crystal

must change as the lattice undergoes changes in packing density as a result of in-plane dynamics (**Supplementary Figure 15**). This modulation of the ionic charge density (while the crystal thickness remains constant) necessarily generates corresponding changes to the electrostatic potential difference, and is the fundamental mechanism through which *p*4-symmetry ^{C98}RhuA crystals are predicted to be piezoelectric. With this property directly confirmed by numerical calculations, we can estimate the electrostatic potential expected analytically for a polar crystal through three distinct (though conceptually similar) methods, using the open-state conformation as an example.

Method 1: Analytical potential predicted by trans-lattice ion distribution. Gauss's law enables calculation of the electric field arising from a given distribution of electric charge. In the presence of a static electric field (generated by the polarized crystal), the distribution of mobile solution ions (free charges) should generate an equivalent field of opposite magnitude at equilibrium. From the "discrete dipoles" (and all-atom MD) we can anticipate Δq = 40*e* for all conformations of a 2×2 unit cell. In the case of an open-state crystal, the cell dimensions are 20×20 nm^2, resulting in a surface charge density of free charges $(\sigma_f) = \pm 0.05 \frac{e}{nm^2} = \pm 8.01 \times 10^{-21} \frac{C}{nm^2}$ on each face of the crystal. Such a configuration allows the system to be modeled as an idealized parallel-plate capacitor of surface charge density $\pm\sigma_f$, separated by a distance d = 5 nm (the thickness of a *p*4 RhuA crystal), and mean-field dielectric with permittivity ε. Under these assumptions, the electric field between the plates is constant, and the voltage (from "free charges"; ΔV_f) between the plates can be calculated as $\Delta V_f = \mathbf{E}_f \times d = \frac{\sigma_f}{\varepsilon\varepsilon_0} \times d$. Conservatively assuming a permittivity corresponding to pure water (ε = 78), we obtain the electric field $\mathbf{E}_f = \frac{\sigma_f}{78\varepsilon_0} = 11.60 \frac{mV}{nm}$ and corresponding voltage: $\Delta V_f = \mathbf{E}_f \times 5\, nm = 57.99\, mV$.

Method 2: Analytical potential predicted from bound surface charge density. The generalized formulation of Gauss's law in Maxwell's equations relates the electric displacement field **D** (arising from both "free" and "bound" charges) to the electric field **E** and density of dipole moments **P** (polarization density) within a material as: $\mathbf{D} = \varepsilon\varepsilon_0\mathbf{E} + \mathbf{P}$. Above, we neglected **P** (no bound charges) to calculate $\mathbf{E}_f$ from the free charge displacement field $\mathbf{D}_f$ (= σ_f). Here, we instead consider only the ^{C98}RhuA macrodipole polarization density **P** for a 2×2 open-state crystal (four proteins in a 20×20×5 nm^3 volume) as $\mathbf{P} = \frac{N\mu}{vol.} = \frac{4 \times 1200\, D}{20{\times}20{\times}5\, nm^3} \times \frac{3.33564^{-30}\, \frac{C \cdot m}{D}}{10^{-27} \frac{m^3}{nm^3}} = 0.008\, \frac{C}{m^2}$. We can then write an expression for the bound surface charge density (σ_b) of on each side of the crystal as the dot product of the polarization density and z axis. As all ^{C98}RhuA dipoles within the lattice are oriented perpendicular to the surface normal we obtain the "bound" surface charge density $\sigma_b = \mathbf{P} \cdot \hat{\boldsymbol{z}} = \pm 0.008\, \frac{C}{m^2}$ on each face of the crystal, identical to value obtained from the trans-lattice ion distribution (σ_f). We then apply the same parallel-plate capacitor model to calculate the "bound" surface charge potential: $\Delta V_b = \mathbf{E}_{\boldsymbol{b}} \times d = \frac{\sigma_b}{\varepsilon\varepsilon_0} \times 5\, nm = -57.99\, mV$. The bound potential V_b is of equal magnitude to V_f, but opposite sign, due to the opposing orientations of the dipolar bound charge density relative to the free charges (see below).

Method 3: Analytical potential predicted from volumetric polarization density. We can alternatively obtain V_b directly from the volumetric polarization density **P**. Now neglecting free charges (**D** = 0) to calculate the field arising from the bound protein dipoles (E_b), we rearrange Gauss's law as $\varepsilon\varepsilon_0\mathbf{E}_{\boldsymbol{b}} = -\mathbf{P}$. Under the same assumptions as above, the "bound charge" voltage is: $\Delta V_b = \mathbf{E}_{\boldsymbol{b}} \times d = -\frac{\mathbf{P}}{\varepsilon\varepsilon_0} = -57.99\, mV$.

The very close agreement between the values for Δq and ΔV determined analytically (40e, −57.99 mV) and numerically from all-atom MD (37.63 ± 9.22e, −56.10 ± 5.71 mV) for open-state $p4$ crystals (**Supplementary Figure 15**) strongly suggests that the predicted macroscale electrochemical properties of ^{C98}RhuA crystals can be effectively estimated from the polarization induced by the oriented macrodipoles of individual RhuA proteins. Importantly, this close agreement is observed for all conformations simulated, demonstrating clear predictive ability from analytical expressions for electric dipoles. We also note that the values provided here conservatively assume a uniform dielectric constant corresponding to pure water. These values could be larger were an effective dielectric constant (*e.g.*, a statistical average of protein and solvent) utilized instead.

Supplementary References

1 Tang, Z., Zhang, Z., Wang, Y., Glotzer, S. C. & Kotov, N. A. Self-Assembly of CdTe Nanocrystals into Free-Floating Sheets. *Science* **314**, 274-278, (2006).

2 Sinyagin, A. Y., Belov, A., Tang, Z. & Kotov, N. A. Monte Carlo Computer Simulation of Chain Formation from Nanoparticles. *J. Phys. Chem. B* **110**, 7500-7507, (2006).

3 Talapin, D. V., Shevchenko, E. V., Murray, C. B., Titov, A. V. & Král, P. Dipole−Dipole Interactions in Nanoparticle Superlattices. *Nano Lett.* **7**, 1213-1219, (2007).

4 Dandekar, P., Kuvadia, Z. B. & Doherty, M. F. Engineering Crystal Morphology. *Annu. Rev. Mater. Res.* **43**, 359-386, (2013).

5 Chung, H. & Diao, Y. Polymorphism as an emerging design strategy for high performance organic electronics. *J. Mater. Chem. C* **4**, 3915-3933, (2016).

6 Joshi, T. *et al.* Local Electronic Structure of Molecular Heterojunctions in a Single-Layer 2D Covalent Organic Framework. *Adv. Mater.* **31**, 1805941, (2019).

7 Hinchet, R., Khan, U., Falconi, C. & Kim, S.-W. Piezoelectric properties in two-dimensional materials: Simulations and experiments. *Mater. Today* **21**, 611-630, (2018).

8 Brant, D. A. & Flory, P. J. The Role of Dipole Interactions in Determining Polypeptide Configurations. *J. Am. Chem. Sci.* **87**, 663-664, (1965).

9 Gilson, M. K., Rashin, A., Fine, R. & Honig, B. On the calculation of electrostatic interactions in proteins. *J. Mol. Biol.* **184**, 503-516, (1985).

10 Gilson, M. K. & Honig, B. Destabilization of an alpha-helix-bundle protein by helix dipoles. *Proc. Natl. Acad. Sci. U.S.A.* **86**, 1524, (1989).

11 Lockhart, D. & Kim, P. Electrostatic screening of charge and dipole interactions with the helix backbone. *Science* **260**, 198-202, (1993).

12 Katz, B. A. & Kossiakoff, A. The crystallographically determined structures of atypical strained disulfides engineered into subtilisin. *J. Biol. Chem.* **261**, 15480-15485, (1986).

13 Tao, J. *et al.* Control of Calcium Phosphate Nucleation and Transformation through Interactions of Enamelin and Amelogenin Exhibits the "Goldilocks Effect". *Cryst. Growth. Des.* **18**, 7391-7400, (2018).
